# The role of insulin as a key regulator of seeding, proliferation, and mRNA transcription of human pluripotent stem cells

**DOI:** 10.1186/s13287-019-1319-5

**Published:** 2019-07-29

**Authors:** Mohammad Shahbazi, Paige Cundiff, Wenyu Zhou, Philip Lee, Achchhe Patel, Sunita L. D’Souza, Fahim Abbasi, Thomas Quertermous, Joshua W. Knowles

**Affiliations:** 1 Stanford Cardiovascular Medicine and Cardiovascular Institute, Stanford School of Medicine, Stanford University, Falk CVRC, Room CV273, MC 5406 300 Pasteur Drive, Stanford, CA 94305 USA; 2 Department of Developmental and Regenerative Biology, Icahn School of Medicine at Mount Sinai, Mount Sinai, New York, NY 10029 USA; 3 Stanford Center for Genomics and Personalized Medicine, Stanford University, Stanford, CA 94305 USA; 4 Stanford Diabetes Research Center, Stanford University, Stanford, CA 94305 USA; 5 Genetics Bioinformatics Service Center, Stanford University, Stanford, CA 94305 USA

**Keywords:** Insulin, Insulin signaling, Proliferation, Apoptosis, Pluripotent stem cells, Transcriptional regulation

## Abstract

**Background:**

Human-induced pluripotent stem cells (hiPSCs) show a great promise as a renewable source of cells with broad biomedical applications. Since insulin has been used in the maintenance of hiPSCs, in this study we explored the role of insulin in culture of these cells.

**Methods:**

We report conditions for insulin starvation and stimulation of hiPSCs. Crystal violet staining was used to study the adhesion and proliferation of hiPSCs. Apoptosis and cell cycle assays were performed through flow cytometry. Protein arrays were used to confirm phosphorylation targets, and mRNA sequencing was used to evaluate the effect of transcriptome.

**Results:**

Insulin improved the seeding and proliferation of hiPSCs. We also observed an altered cell cycle profile and increase in apoptosis in hiPSCs in the absence of insulin. Furthermore, we confirmed phosphorylation of key components of insulin signaling pathway in the presence of insulin and demonstrated the significant effect of insulin on regulation of the mRNA transcriptome of hiPSCs.

**Conclusion:**

Insulin is a major regulator of seeding, proliferation, phosphorylation and mRNA transcriptome in hiPSCs. Collectively, our work furthers our understanding of human pluripotency and paves the way for future studies that use hiPSCs for modeling genetic ailments affecting insulin signaling pathways.

**Electronic supplementary material:**

The online version of this article (10.1186/s13287-019-1319-5) contains supplementary material, which is available to authorized users.

## Background

Human-induced pluripotent stem cells (hiPSCs) can be expanded continuously and can differentiate into all lineages of the human body. Due to these characteristics, hiPSCs provide a renewable source of cells for developmental studies, drug screening, and regenerative medicine [1]. Additionally, undifferentiated hiPSCs can recapitulate the aspects of genetic diseases provided that the affected cellular and molecular pathways are active in the pluripotent cells [2–4]. Since the initial description of human-induced pluripotent stem cells [5, 6], there has been a growing interest in identification of essential components of their culture. Studying these key components and the respective signaling pathways improves our understanding of pluripotency and also demonstrates the potentials and limitations of hiPSCs in modeling of diseases associated with cell signaling.

Transforming growth factor beta 1 (TGFB1) and fibroblast growth factor 2 (FGF2) are two of the components that have been identified that seem to be essential in the maintenance of human pluripotent stem cells. Another essential component is insulin, a peptide hormone that regulates an intricate network of signaling pathways and cellular responses [7] and has been identified as one of the key ingredients in culture of human pluripotent stem cells [8–10]. However, the role of insulin in the culture of hiPSCs is not fully understood. In this study, we report conditions for insulin starvation of hiPSCs for up to 3 days and use this setting to explore the effect of insulin on several cellular and molecular functions that have been associated with insulin signaling as well as explore the effect of insulin on global mRNA transcription in hiPSCs.

## Methods

### Culture of hiPSCs

hiPSC lines were derived from PBMCs using integration-free Sendai virus to deliver the Yamanaka factors as reported earlier [11]. These cell lines were initially expanded on matrigel in the presence of mTeSR1 (StemCell Technologies) and passaged using 0.5 mM EDTA. All cells were then thawed and adapted to TeSR-E8 for at least 2 weeks prior to the experiments. The medium was changed daily starting within the initial 3 days of culture. To prepare an insulin-free hPSC medium, mTeSR-E5-defined medium (StemCell Technologies) was supplemented with FGF2 (bFGF, PeproTec, final concentration of 100 μg/l) and TGFB1 (PeproTec, final concentration of 2 μg/l). Cells were washed 4 times with DMEM-F12 (Life Technologies) prior to insulin starvation experiments. When insulin treated groups were needed for the experiments, the medium was further supplemented with insulin (insulin, human recombinant, zinc solution; Life Technologies) to the final concentration of 19.4 mg/l. During maintenance in TeSR-E8 and experiments, the cultures were fed double medium from day 4 of culture onwards, unless the cells were used for overnight experiments on day 4.

Of the four hiPSC lines initially used in the preliminary experiments (Additional file 4: Table S1), one line showed sporadic patches of differentiating colonies (data not shown). While the vast majority of the cells maintained their pluripotent morphology in the line, we excluded this line from our experiments to prevent the chance that traces of differentiated cells interfere with our data analysis.

### Immunofluorescence staining

Primary and secondary antibodies used for pluripotency were obtained from a Pluripotent Stem Cell 4-Marker Immunocytochemistry Kit (Life Technologies). DAPI was used for counter staining of the nuclei. Secondary antibody stainings were used as controls (data not shown).

### Crystal violet staining

Crystal violet staining was used for visualization of the cells in quantification of cell adhesion and cell expansion. Samples were fixed with 4% PFA in PBS for 5 min and were stained with 0.05% crystal violet solution for 30 min; plates were rinsed up to 4 times with water, dried, and were used for imaging. The staining in the wells was quantified using microscopic imaging of the wells for seeding experiments, or direct images of the wells for expansion experiments. The percentage of surface area covered by cells (stained regions) was measured using photoshop and was compared between groups.

### Cell cycle assay

Cells were harvested with accutase (Millipore) or Stempro Accutase (Life Technologies), fixed with 70% ethanol and stained through incubation of cells in PBS-0.1% Triton-X solution supplemented with 0.2 mg/ml RNase A and 0.02 mg/ml propidium iodide (PI) at room temperature for 30 min. Prepared samples were analyzed through flow cytometry. FlowJo software (Treestar, Inc) was used for modeling and analysis of the cell cycle.

### Apoptosis assay

Cells were harvested with accutase. Supernatant and rinse solutions were collected separately and were pooled with the harvested cells prior to staining. Samples were stained using dead cell apoptosis kit with Annexin V FITC and PI (Life Technologies) and analyzed through flow cytometry.

### Protein arrays

Human Phospho-RTK Antibody Array, Human Phospho-MAPK Antibody Array, Human Phospho-Kinase Antibody Array, and Human Apoptosis Antibody Array Kits were obtained from R&D system and used according to the provided instructions. Briefly, lysates were harvested after treatment of cells in the described experimental conditions. The cells were rinsed once with PBS (with Ca/Mg) and lysed with inhibitors supplemented with lysis buffer. Concentration of protein lysates were subsequently determined by BCA assay. The membranes were incubated overnight with protein lysate at 4 °C on a rocking platform before incubation with a secondary antibody or directly with a detection reagent based on the array manual provided with the kit.

Li-Cor Odyssey imager and Image Studio software (Li-Cor Biosciences) were used for imaging and quantification of protein array results. Image Studio software was used to quantify the signal of the array dots. The data were normalized in each dataset through division of signal value obtained from groups treated without insulin to their respective insulin-treated groups.

### RNA extraction and RNA sequencing

RNA extraction and RNA sequencing were performed as described earlier (Carcamo-Orive et al., 2017). Sequencing reads were first quality checked by FastQC (v 0.11.8). Reads above Q30 were further mapped and reads per gene were counted by STAR (v2.7). Read count normalization and differential analyses were performed using Deseq2 R package (v1.22.2) with custom codes. Pathway enrichments were performed using Gene Set Enrichment Analysis (GSEA) with MSigDB 6.2 database. For 24 h, all significantly (*p*_adj < 0.05) differential genes were included for the enrichment analysis, while for 72 h, the significantly (*p*_adj < 5E10–4) differential genes were included to obtain a similar number of significant genes as compared to those under 24 h for the enrichment analysis.

## Results

### The effect of insulin on seeding of hiPSCs

**Fig. 1:**
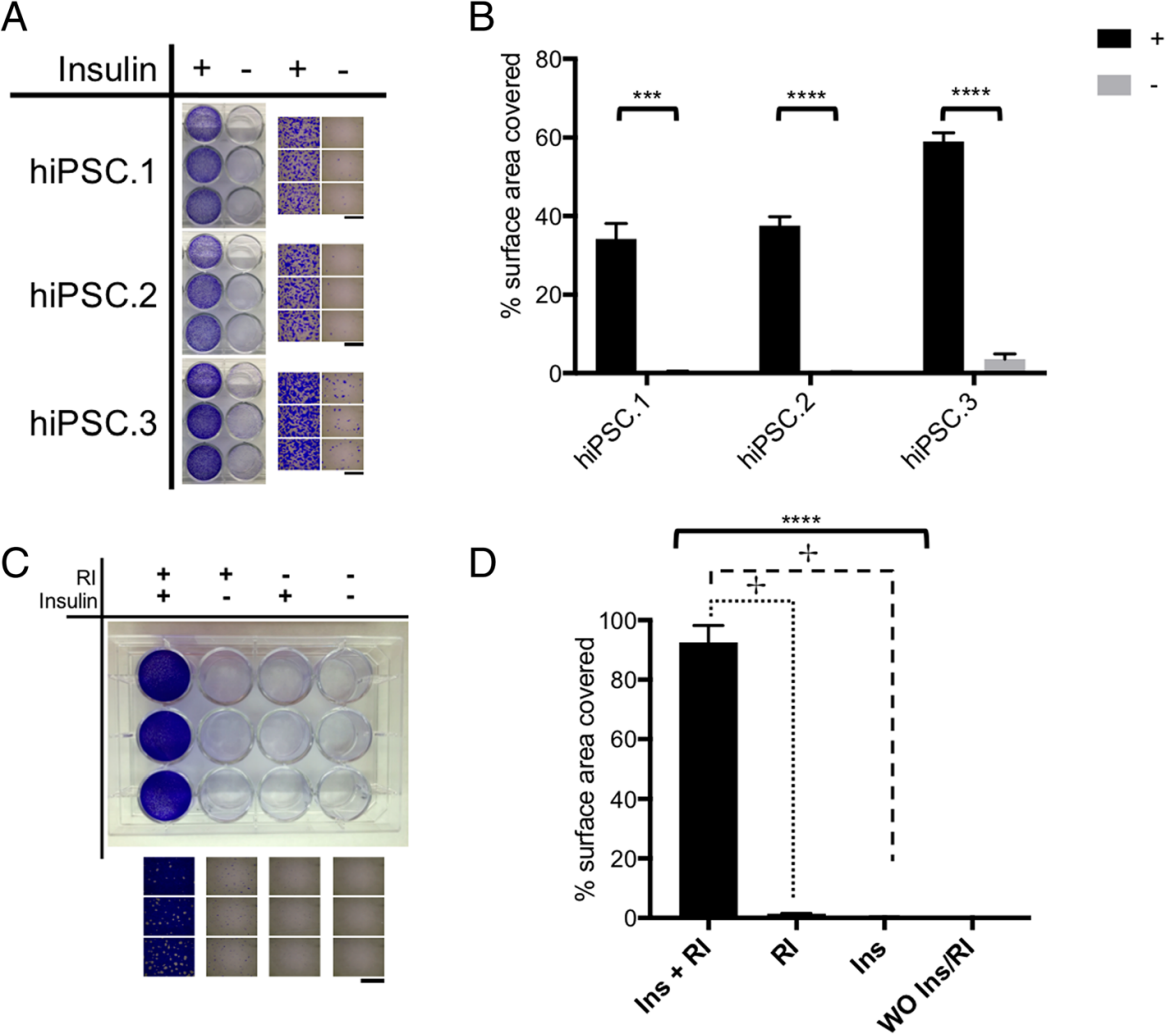
The effect of insulin on seeding of hiPSCs. hiPSCs were harvested with EDTA as cell aggregates and were imaged 1 day after seeding. **a** Whole-well images and microscopic images from the center of each well after crystal violet staining. **b** Quantification of the percentage surface area covered by hiPSC fragments for the microscopic images from **a**. Data represented as mean ± SD. Two-tailed Student’s *t* test was used for comparison of groups, each cell line in triplicates. hiPSC.3 colonies were harvested with accutase as single cells/small cell fragments and were passed through a 40-μm filter. **c** Whole-well images and microscopic images of the center of each well after crystal violet staining. **d** Quantification of percentage of surface area covered by hiPSCs for the microscopic images from **c**. Data represented as mean ± SD. One-way ANOVA test was used between first three groups; no adherent cells were detected in the last group. ***p < 0.001. ****p < 0001. ✢Significant difference between groups in LSD test. Experiment in triplicates. Scale bar: 2 mm. RI: rock inhibitor

To investigate the effect of insulin on seeding of hiPSC aggregates, colonies were harvested by EDTA and were seeded with or without insulin. The attachment of the hiPSC aggregates was evaluated microscopically after crystal violet staining of plates 1 day after seeding. The presence of insulin significantly improved the seeding of hiPSC aggregates (Fig. 1a, b).

To determine the effect of insulin on seeding of dissociated hiPSCs, the colonies were digested with Accutase and passed through the 40-μM cell strainer and were used for crystal violet staining. The presence of Y-27632, which is an inhibitor of rho-associated kinase (ROCK), prevents apoptosis in dissociated hiPSCs [12]. Our results demonstrate that the presence of both Y-27632 and insulin are essential for attachment of dissociated hiPSCs (Fig. 1c, d). Inclusion of either component allowed very limited attachment of cells while no attached cells were observed when both reagents were absent.

### The effect of insulin on pluripotent morphology, expression of pluripotency markers, and expansion of hiPSC colonies

**Fig. 2:**
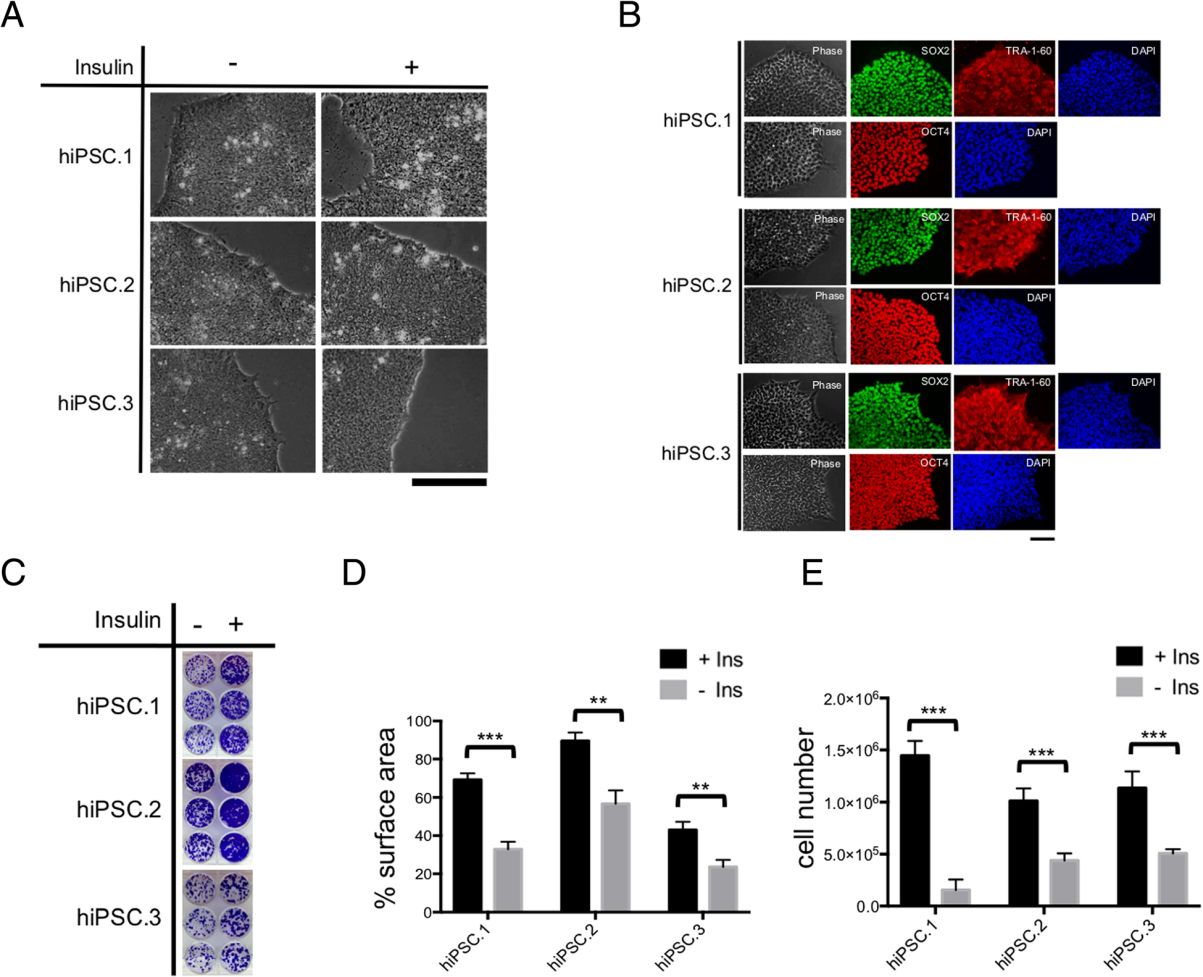
The effect of insulin on pluripotency phenotype and expansion of hiPSC colonies. hiPSC colonies maintain both their pluripotent. Morphology by phase contrast (**a**) and expression of key pluripotency markers, SOX2, OCT4 (also known as POU5F1), and TRA-1-60 (**b**) after 3 days of insulin starvation. Scale bar, 100 μM. Whole-well images (each cell line in triplicates) were taken after crystal violet staining (**c**) and quantification of surface area covered by hiPSC (**d**). The number of cells (each cell line in triplicates) in each culture condition was determined through cell count (**e**). Data represented as mean ± SD. Two-tailed Student’s *t* test was used for comparison of groups

To compare the effect of insulin on pluripotent morphology and expression of pluripotency markers, 3 lines of hiPSCs (Additional file 4: Table S1) were maintained in culture for 3–4 days and were subsequently cultured with or without insulin for 3 additional days. These cells maintained pluripotent morphology (Fig. 2a) and expression of key pluripotency markers SOX2, POU5F1, and TRA-1-60 after 3 days of insulin starvation (Fig. 2b).

The surface area of the tissue culture plate covered by hiPSC colonies in the presence of insulin was significantly larger than the surface area covered in the absence of insulin (Fig. 2c, d). These observations were in line with number of cells in each condition, determined by manual cell count (Fig. 2e) and indicate that the presence of insulin significantly improves the proliferation of hiPSCs.

### Insulin changes the cell cycle profile and inhibits apoptosis in hiPSCs

**Fig. 3:**
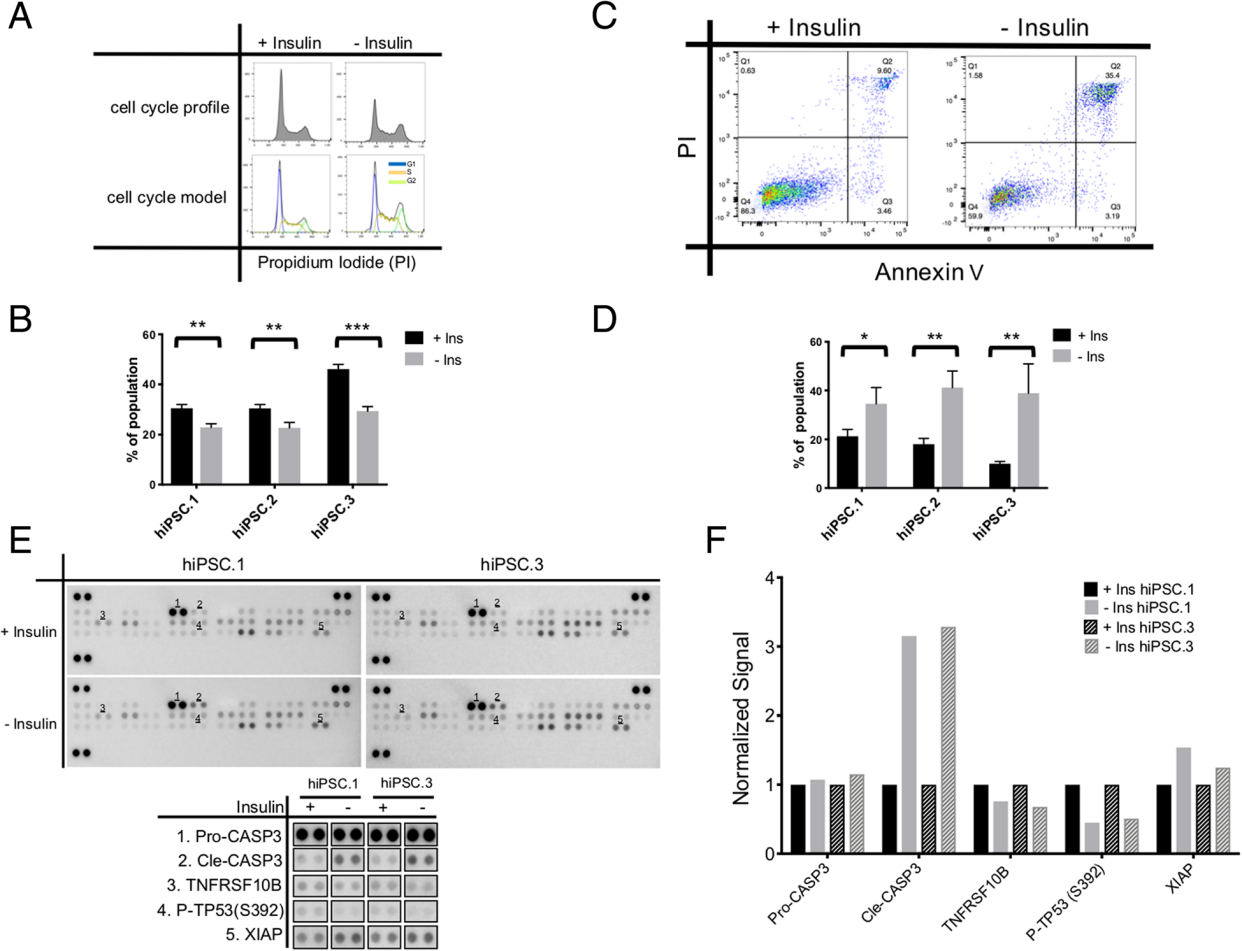
The effect of insulin on cell cycle and apoptosis of hiPSCs. To investigate the effect of insulin on cell cycle of hiPSCs, the cells were cultured with or without insulin for 3 days and used for cell cycle assay. **a** A representative histogram of change in the cell cycle profile (upper panel) and the model (lower panel). **b** Quantification of percentage of cells at the G1 stage (each cell line in triplicates). To investigate the effect of insulin on apoptosis of hiPSCs, the cells were cultured with or without insulin overnight and used for flow cytometry-based apoptosis assay (hiPSC.1 in quintuplicate, hiPSC.2 in triplicates, hiPSC.3 in quadruplicate) or protein array for apoptosis. **c** Representative graphs of change in cell apoptosis profile. **d** Quantification of percentage of cells in late apoptosis stage (high PI and high Annexin V). **e** Protein array apoptosis array, targets and their quantification (**f**). Data in **b** and **d** are represented as mean ± SD. Two-tailed Student’s *t* test was used for comparison of groups. *p < 0.05, **p < 0.01, ***p < 0.001

To investigate the effect of insulin on cell cycle, the cells were cultured with or without insulin for 3 days and then used for flow cytometry-based cell cycle assay using propidium iodide (PI) staining. There was a significant decrease in the percentage of the cells at the G1 phase is the absence of insulin (Fig. 3a, b).

After hiPSCs were cultured overnight (16–18 h) with and without insulin, there was a significant increase in detected apoptotic cells in the absence of insulin in a flow cytometry-based apoptosis assay (Fig. 3c, d).

Protein array analysis of apoptotic proteins after 16 h of insulin starvation demonstrates more than a 3-fold increase in levels of cleaved caspase-3 while levels of pro-caspase-3 remain at similar levels (Fig. 3e, f). This robust increase in levels of cleaved caspase-3 is accompanied by reduction in XIAP, TNFRSF10B proteins, and serine 392 phosphorylated TP53.

### Verification of insulin-mediated protein phosphorylation in hiPSCs

**Fig. 4:**
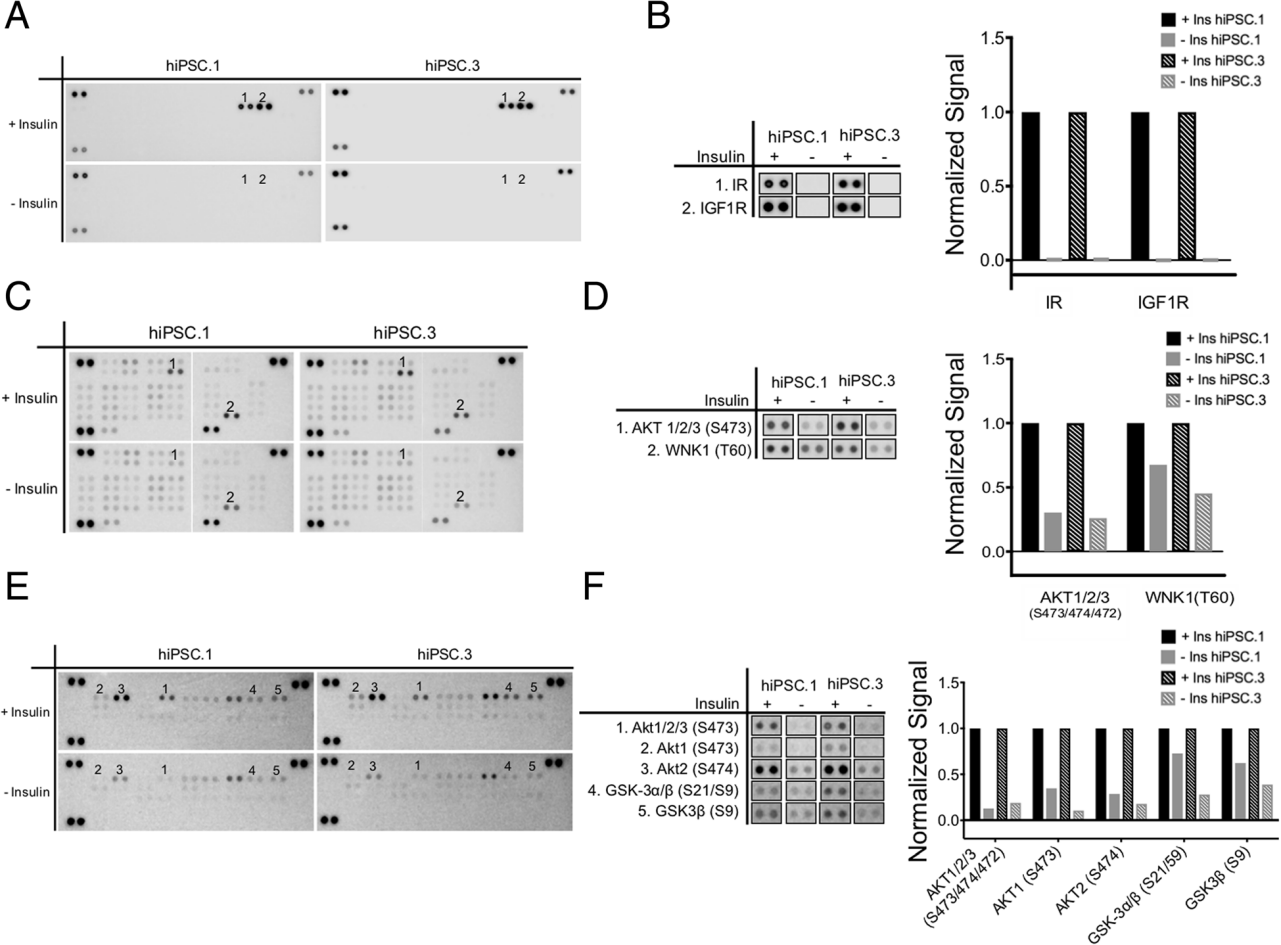
The effect of insulin on phosphorylation of RTKs, HPKs, and MAPKs. To investigate the effect of insulin on phosphorylation of target proteins in hiPSCs, the cells were cultured with or without insulin overnight followed by 15-min stimulation with insulin prior to harvesting the lysates for protein arrays. **a** Receptor tyrosine kinase (RTK) array. **b** Identified targets from RTK array and their respective quantification. **c** Human phosphokinase (HPK) array. **d** Identified targets from HPK array and their quantification. **e** Human phospho MAP kinase(MAPK) array. **f** Identified targets from MAPK array and their quantification

We used protein arrays to monitor phosphorylation of a set of tyrosine kinase receptors and phosphokinases (Fig. 4). We confirmed that the insulin receptor (INSR) and insulin-like growth factor 1 receptor (IGF1R) are highly phosphorylated in the presence of insulin. Furthermore, our results indicate that phosphorylation of AKT and WNK1 and GSK3 are regulated by insulin stimulation in hiPSCs.

### Insulin regulates transcriptome of pluripotent stem cells

To gain an understanding of the transcriptional changes induced by insulin in hiPSCs, we looked at the effect of insulin on gene expression at 24 h and 72 h.

An average of 62.7 million reads were obtained for each sample, and the average genome mapping rate was 90% (Additional file 1: Figure S1 and Additional file 2: Figure S2). The number of identified genes is shown in Additional file 4: Table S2.

**Fig. 5:**
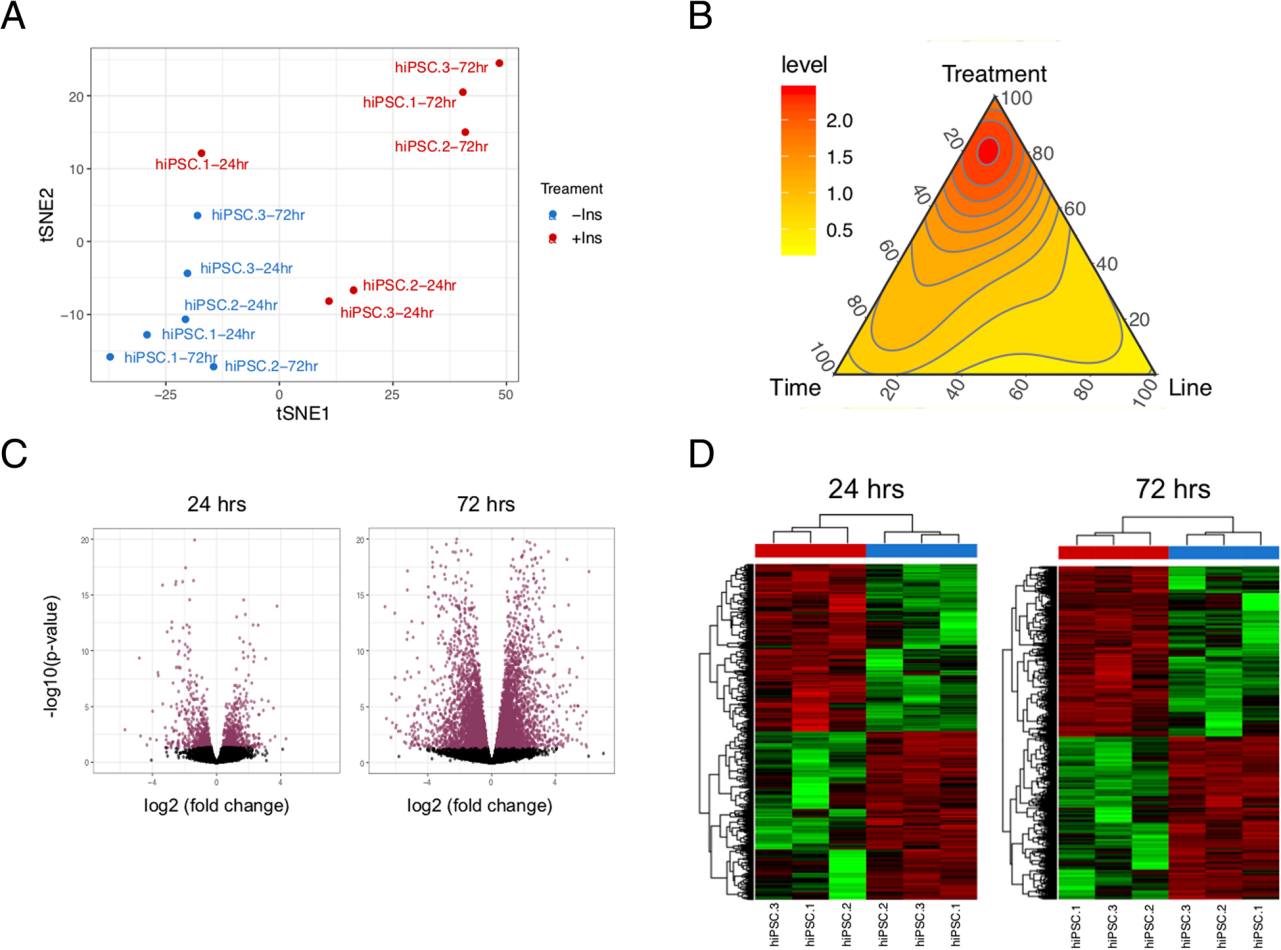
The effect of insulin on mRNA transcriptome of hiPSCs. **a** T-distributed Stochastic Neighbor Embedding (t-SNE) plot for all the samples with or without insulin. **b** Ternary plot showing density distribution of the percentage of variances explained by the three major variables, namely treatment (+/− insulin), time (24 h or 72 h), and cell line (hiPSC.1, hiPSC.2, hiPSC.3). For both t-SNE and variance decomposition analysis, all 21,575 mapped genes were used. **c** The relationship between the log2 fold change and *p* values in normalized read counts per gene is shown in volcano plots. Genes with a *q* value < 0.05 are shown in red which are 1161 genes in 24 h and 6753 genes in 72 h time points. **d** Heatmaps of differentially expressed genes at each time point. Both rows and columns are clustered by unsupervised clustering based on their Manhattan distance

The prominent effect of insulin on global mRNA transcription of the cells is demonstrated by the t-SNE plot (Fig. 5a) and ternary plot (Fig. 5b). The t-SNE plot clearly indicates that the presence of insulin has a stronger global effect on transcription than internal variations across the cells at 72 h. This is manifested by closer clustering of samples based on insulin treatment rather than the individual lines at 72 h time point. A ternary plot shows that among the three major variables (time, cell line, or treatment), insulin treatment explains most of the variances at a majority of the mapped genes.

We identified 1161 differentially expressed genes at 24 h of hiPSC culture with and without insulin and 6753 differentially expressed genes at 72 h, with *q* value < 0.05. The 20 top differentially expressed genes at 24 h and 72 h time points are listed in Additional file 4: Tables S3 and S4. The relationship between log2 fold change and *p* values is shown as volcano plots for each time point with differentially expressed genes shown in red (Fig. 5c). The heatmaps of differentially expressed genes at 24 h and 72 h are shown in Fig. 5d.

**Fig. 6:**
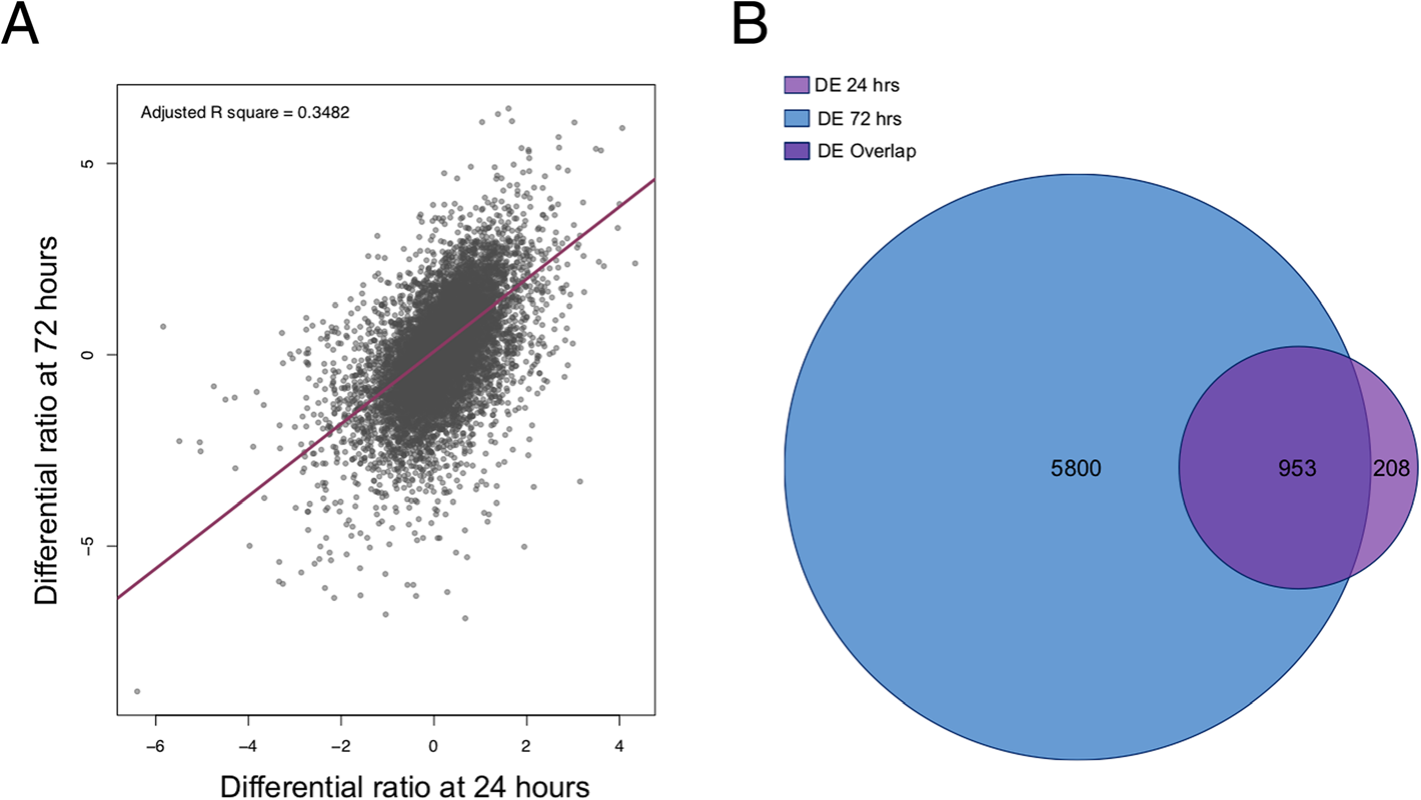
Comparison of gene expression at 24 and 72 h of hiPSC culture with or without insulin. **a** Scatter plot showing the correlation between the log2 fold change (+ insulin/− insulin) at 24 h versus 72 h, with a *p* value < 2.2e−16. All genes were included for this comparison. **b** The number of overlapping and unique genes that were differentially expressed between the two time points by Venn diagram

We compared the effect of insulin on gene expression at 24 h and 72 h. There is a strong correlation between gene expression at 24 h and 72 h suggesting that the effect of insulin on transcriptome is generally consistent between 24 h and 72 with more prominent effect at 72 h (Fig. 6a). This consistency is also demonstrated by overlap of genes differentially expressed at 24 h with 72 h (Fig. 6b).

To better understand the affected biological processes using identified differentially expressed genes, we performed Gene Ontology (GO) term enrichment for biological processes at each time point.

The top 25 terms that were associated with differentially expressed transcripts at 24 h are shown in Table 1. The top 25 biological Gene Ontology (GO) processes terms that were associated with significantly differentially expressed transcripts at 72 h are shown in Additional file 4: Table S5. From the list of top 20 GO processes at 24 h, we selected the high-ranked terms associated with our results and heatmaps of differentially expressed genes within each term were generated (Additional file 3: Figure S3).

**Table 1:** The top 25 significant GO (biological process) terms associated differentially expressed transcripts at 24 h of hiPSC culture with or without insulin

	GO term	# Genes in gene set (K)	# Genes in overlap (k)	k/K	*p* value	FDR *q* value
1	GO_REGULATION_OF_INTRACELLULAR_SIGNAL_TRANSDUCTION	1656	148	0.0894	7.93E−48	3.52E−44
2	GO_TISSUE_DEVELOPMENT	1518	130	0.0856	6.17E−40	1.37E−36
3	GO_CELL_DEVELOPMENT	1426	125	0.0877	2.05E−39	3.03E−36
4	GO_REGULATION_OF_PHOSPHORUS_METABOLIC_PROCESS	1618	133	0.0822	5.80E−39	6.43E−36
5	GO_POSITIVE_REGULATION_OF_RESPONSE_TO_STIMULUS	1929	139	0.0721	1.52E−34	1.35E−31
6	GO_RESPONSE_TO_EXTERNAL_STIMULUS	1821	134	0.0736	3.23E−34	2.39E−31
7	GO_REGULATION_OF_CELL_PROLIFERATION	1496	120	0.0802	4.09E−34	2.59E−31
8	GO_POSITIVE_REGULATION_OF_MULTICELLULAR_ORGANISMAL_PROCESS	1395	115	0.0824	8.90E−34	4.93E−31
9	GO_NEUROGENESIS	1402	115	0.082	1.41E−33	6.93E−31
10	GO_INTRACELLULAR_SIGNAL_TRANSDUCTION	1572	122	0.0776	2.60E−33	1.15E−30
11	GO_POSITIVE_REGULATION_OF_CELL_COMMUNICATION	1532	120	0.0783	3.85E−33	1.55E−30
12	GO_REGULATION_OF_CELL_DEATH	1472	117	0.0795	6.77E−33	2.50E−30
13	GO_REGULATION_OF_MULTICELLULAR_ORGANISMAL_DEVELOPMENT	1672	125	0.0748	1.46E−32	4.98E−30
14	GO_RESPONSE_TO_ENDOGENOUS_STIMULUS	1450	115	0.0793	3.00E−32	9.51E−30
15	GO_CIRCULATORY_SYSTEM_DEVELOPMENT	788	83	0.1053	7.93E−32	2.35E−29
16	GO_PHOSPHATE_CONTAINING_COMPOUND_METABOLIC_PROCESS	1977	135	0.0683	3.57E−31	9.89E−29
17	GO_REGULATION_OF_PROTEIN_MODIFICATION_PROCESS	1710	124	0.0725	4.79E−31	1.25E−28
18	GO_REGULATION_OF_TRANSPORT	1804	127	0.0704	1.44E−30	3.55E−28
19	GO_MOVEMENT_OF_CELL_OR_SUBCELLULAR_COMPONENT	1275	104	0.0816	3.45E−30	8.05E−28
20	GO_POSITIVE_REGULATION_OF_GENE_EXPRESSION	1733	123	0.071	6.25E−30	1.39E−27
21	GO_CELLULAR_RESPONSE_TO_ORGANIC_SUBSTANCE	1848	127	0.0687	1.41E−29	2.98E−27
22	GO_POSITIVE_REGULATION_OF_MOLECULAR_FUNCTION	1791	124	0.0692	3.56E−29	7.18E−27
23	GO_ENZYME_LINKED_RECEPTOR_PROTEIN_SIGNALING_PATHWAY	689	73	0.106	2.96E−28	5.70E−26
24	GO_RESPONSE_TO_OXYGEN_CONTAINING_COMPOUND	1381	105	0.076	5.68E−28	1.05E−25
25	GO_REGULATION_OF_CELLULAR_COMPONENT_MOVEMENT	771	76	0.0986	2.37E−27	4.20E−25

We compared differentially expressed genes at 24 h and 72 h with KEGG pathway database to investigate the effect of insulin on expression of known genes involved in pathways regulating the pluripotency (Additional file 4: Table S6) and insulin signaling pathway (Additional file 4: Table S7).

## Conclusion

We investigated the effect of insulin on seeding of hiPSC aggregates or dissociated hiPSCs. Our results with dissociated hiPSCs demonstrate that insulin exerts its effect on their seeding independent of Y-27632. This observation is in line with an earlier report indicating that insulin is essential for survival of dissociated human pluripotent stem cells [8]. We expand on this by also demonstrating the significant effect of insulin on seeding of hiPSC aggregates without use of either chemical inhibitors blebbistatin or Y-27632.

Our data indicate that insulin significantly improves the expansion of hiPSC colonies and cellular proliferation. These results are broadly in line with earlier reports [8], although we observe less drastic effect after insulin starvation in our experiments on the expansion of the cells, possibly because Chen et al. used dissociated cells rather than hiPSC aggregates in their experiments. In our work, we also investigated the effect of insulin in expansion of hiPSC colonies without using of blebbistatin or Y-27632 and separated the seeding experiments from expansion experiments.

Specific inhibition of IGF1R with shRNA in human embryonic stem cells (hESCs) has been reported to lead to their competitive disadvantage in proliferation [10], demonstrating the importance of IGF1R in expansion of human pluripotent stem cells. In addition, hiPSCs derived from insulin-resistant patients with mutations in their insulin receptor have demonstrated lower proliferation compared to their normal counterparts in the presence of insulin [2], suggesting that insulin signaling plays a regulatory role in proliferation of hiPSCs. Phosphorylation of IGF1R and INSR in the presence of conditioned medium containing high levels of insulin [10] or after insulin stimulation [2] has also been reported. Consistent with these studies, we observed robust phosphorylation of both the INSR as well as IGF1R. In addition, our data appear to demonstrate a more robust phosphorylation of these receptors, potentially due to different starvation conditions or higher concentrations of insulin used.

Cell division and cell death are two main contributory factors to cellular proliferation. We observed a significant increase in apoptosis by hiPSCs in the absence of insulin. This observation was consistent with the detection of higher levels of cleaved caspase-3. Caspase-3 plays a key role in execution stage of apoptosis. Interestingly, cleavage of pro-caspase-3 has been reported after inhibition of AKT phosphorylation in hiPSCs [13]. In addition, we detected lower levels of TNFRSF10B after insulin starvation. TNFRSF10B has been reported to play a dual role in cell survival [14].

After insulin starvation, we detected lower levels of serine 392 phosphorylated TP53 and higher level of XIAP in an apparent contrast to the usual role attributed to them. Serine 392 phosphorylated TP53 has been reported to contribute to genotoxic stress and apoptosis [15]. XIAP has also been identified as an anti-apoptotic molecule; nevertheless, upregulation of XIAP in response to environmental stress stimuli to induce apoptosis resistance has been reported in a cellular model [16]. These observations can be potentially attributed to the coping mechanism in surviving cells in response to the activated apoptotic pathways.

Phosphorylation of AKT is a critical node in insulin signaling [7]. AKTs play a central role in pluripotency and it has been demonstrated that overexpression of AKT improves the maintenance of pluripotent morphology in a primate pluripotent stem cell model [17]. Our data confirms that AKTs including AKT1 and AKT2 are main targets of insulin signaling in hiPSCs, consistent with earlier reports [18]. We also demonstrate that phosphorylation of GSK3B is a target of insulin stimulation in hiPSCs. Inhibition of phosphorylation of both AKT and GSK3B has been demonstrated to increase apoptosis in hiPSCs [13, 19].

Despite phosphorylation of AKT and GSK3B in the presence of insulin, we still detected a basal level of phosphorylation of these target proteins in the absence of insulin. This observation is in line with earlier reports of contribution of other growth factors including endogenously secreted ELABELA [18] and high concentrations of FGF [20] in the medium which contribute independently to activation of AKT signaling pathway in hiPSCs.

With regard to cell cycle profile of hiPSC populations after 3 days of insulin starvation, we observed a significant reduction in percentage of cells in the G1 phase. This change in cell cycle profile is similar to reports regarding inhibition of phosphorylation of either GSK3B (which we here demonstrate to be a target of insulin signaling in hiPSCs) or receptor tyrosine kinase ERBB2 in human pluripotent stem cells. In both studies, inhibition of phosphorylation led to decrease in G1 phase accompanying an increase in apoptosis or decrease in proliferation in these cells [10, 13]. This reduction in percentage of cells in the G1 phase alongside a decrease in the proliferation of the cells suggests a cell cycle delay or arrest at G2/M transition. Our data also demonstrates that WNK1 is strongly phosphorylated upon insulin stimulation in hiPSCs. WNK1 has been identified as a downstream target of phosphorylation by AKT/PKB after insulin and IGF-1 stimulation in other cell lines [21, 22]. The cellular significance of this phosphorylation in the context of hiPSC biology would be an interesting avenue for follow-up studies.

To our knowledge, we are the first group to demonstrate the robust regulatory effect of insulin on mRNA transcriptome of hiPSCs. Insulin regulates expression of wide array of genomic targets [23]. Using our starvation protocol, we explored the effect of insulin on gene expression in pluripotent stem cells after 24 h and 72 h. Our data also indicate that the effect of insulin on the transcriptome at 24 h and 72 h seem to be generally in the same direction while becoming more robust overtime.

At the 24 h time point and as expected, the top 25 identified terms include terms consistent with the role of insulin in cell signaling and transduction. Furthermore, regulation of both cell proliferation and cell death are also among the top 25 identified terms supporting the role of insulin in cell signaling and cellular proliferation of hiPSCs. All these terms remain among the top 100 terms at 72 h.

We compared the differentially expressed genes at 24 h with identified genes in insulin signaling pathway (KEGG pathway). Among the 9 identified genes, 8 are also differentially expressed at 72 h. Among these 8, there are classic components of insulin signaling pathway including INSR, IRS1, IRS2, and CRK. These are expressed at lower levels in the presence of insulin, while signaling suppressor SOCS2 is expressed at higher levels in the presence of insulin. This observation is in line with a negative regulation feedback of components insulin signaling pathway in response to activated insulin signaling or increased sensitivity of hiPSCs cultured without insulin.

We also compared the differentially expressed genes at 24 h with identified genes involved in regulating pluripotency (KEGG pathway). All of the 7 identified differentially expressed genes are also differentially expressed at 72 h. From these genes, FZD2 and DVL3 belong to Wnt signaling pathway and APC2 is a regulator of the Wnt pathway. Expressions of FZD2 and DVL3 are lower while expression of APC2 is higher in the presence of insulin. These observations suggest that insulin might exert a regulatory effect on Wnt signaling pathway in hiPSCs. The role of Wnt signaling in human pluripotent cells remains controversial [24]. An interesting identified gene is LIF, with a higher expression in the presence of insulin. LIF plays a critical role in maintenance of naïve pluripotent stem cells which represent an earlier developmental stage of pluripotency than hiPSCs (primed stage) [25]. LIF has been shown to increase the proliferation in other human cells [26, 27] and is a potential inducer of proliferation in the presence of insulin. Another interesting target is BMP4. Expression of BMP4 appears to be significantly lower in the presence of insulin at both time points. Elevated levels of BMP4 can trigger differentiation of human pluripotent stem cells into mesodermal lineages [28, 29] although differentiation process is suppressed by cell contact [30]. These observations suggest that insulin might maintain hiPSCs at a higher pluripotency stage and less susceptible to differentiation.

In summary, we confirmed that insulin plays a significant role in regulation of proliferation, apoptosis, protein phosphorylation, and mRNA transcription in hiPSCs. Our findings will improve our understanding of the role of insulin in hiPSC biology and are reasonably expected to contribute to better disease modeling experiments affecting the insulin signaling pathways using hiPSCs. We are now focusing on understanding the global transcriptional differences between hiPSCs of insulin-resistant and insulin-sensitive individuals in the presence of insulin.

## Additional files

Additional file 1: **Figure S1.** RNA sequencing Data QC, related to results section regarding RNA sequencing. (A) Average read quality of each sequencing data. The x-axis shows the average read Q-score and the y-axis indicates the number of reads. (B) Base quality along the reads of the sequencing data. The x-axis shows the position in the read is plotted and the y-axis shows the Q-score is plotted. Median value Q-score are shown by the red lines. The mean value Q-score are represented by dark blue line. The inter-quartile range is shown by the boxplot, while the 10% and 90% points are represented by the whiskers. (TIFF 6593 kb)

Additional file 2: **Figure S2.** Mapping results, related to the “Results” section regarding RNA sequencing. Samples’ summary of mapping results of the reads. In each sample reads could be classified into: mapped (aligned to reference genome), high abundance or out-mapped (e.g., rRNA, mtRNA, polyA, polyC) and unmapped (reads which did not align to anything). (TIFF 6593 kb)

Additional file 3: **Figure S3.** Differentially expressed genes in selected GO pathways related to 24 h of hiPSC culture with or without insulin, related to Table 1 regarding the top 25 significant GO (biological process) terms. (TIFF 6593 kb)

Additional file 4: **Table S1.** List of hiPSC lines used. **Table S2.** Number of identified genes in each sample, related to results section regarding RNA sequencing. **Table S3.** The 20 most significantly differentially expressed genes at 24 h of hiPSC culture with or without insulin, related to results section regarding RNA sequencing. The list is ordered by adjusted *p* values. Average read counts of samples with no insulin treatment are shown in the “baseMean” column. Fold change is the log2 fold change of normalized read count per gene between groups. Followed are the lfcSE (log fold change Standard Error) and stat (Wald statistics). Also listed are *p* values and adjusted *p* values corrected for multiple testing using the Benjamini-Hochberg False Discovery Rate (FDR) approach. **Table S4.** The 20 most significantly differentially expressed genes at 72 h of hiPSC culture with or without insulin, related to results section regarding RNA sequencing. The list is ordered by adjusted *p* values. Average read counts of samples with no insulin treatment are shown in the “baseMean” column. Fold change is the log2 fold change of normalized read count per gene between groups. Followed are the lfcSE (log fold change Standard Error) and stat (Wald statistics). Also listed are *p* values and adjusted *p* values corrected for multiple testing using the Benjamini-Hochberg False Discovery Rate (FDR) approach. **Table S5.** The top 100 significant GO (biological process) terms associated with differentially expressed transcripts after 72 h of hiPSC culture with or without insulin, related to results sections regarding RNA sequencing. **Table S6.** Differentially expressed genes from culture of hiPSCs with or without insulin that have been identified as a part of KEGG signaling pathways regulating pluripotency of stem cells (human). **Table S7.** Differentially expressed genes from culture of hiPSCs with or without insulin that have been identified as a part of KEGG insulin signaling pathway (human). (DOCX 74 kb)

## Data Availability

The datasets used and/or analyzed during the current study are available from the corresponding author on reasonable request. RNA-Sequencing data will be available through GEO database.

## Abbreviations

APC2: APC regulator of WNT signaling pathway 2
BMP4: Bone morphogenetic protein 4
DVL3: Disheveled segment polarity protein 3
FGF2: Fibroblast growth factor 2
FZD2: Frizzled class receptor 2
GO: Gene Ontology
hESCs: Human embryonic stem cells
hiPSCs: Human-induced pluripotent stem cells
HPK: Human phosphokinase kinase
IGF1R: Insulin-like growth factor 1 receptor
INSR: Insulin receptor
IRS: Insulin receptor substrate
LIF: Leukemia inhibitory factor
MAPK: Mitogen-activated protein kinase
PI: Propidium iodide
ROCK: Rho-associated kinase
RTK: Receptor tyrosine kinase
SOCS2: Suppressor of cytokine signaling 2
TGFB1: Transforming growth factor beta 1
