# Supplementary figures and images for: The role of insulin as a key regulator of seeding, proliferation, and mRNA transcription of human pluripotent stem cells
Source: Stem Cell Res Ther. 2019 Jul 29;10:228. doi: 10.1186/s13287-019-1319-5 (PMC6664730; doi:10.1186/s13287-019-1319-5)

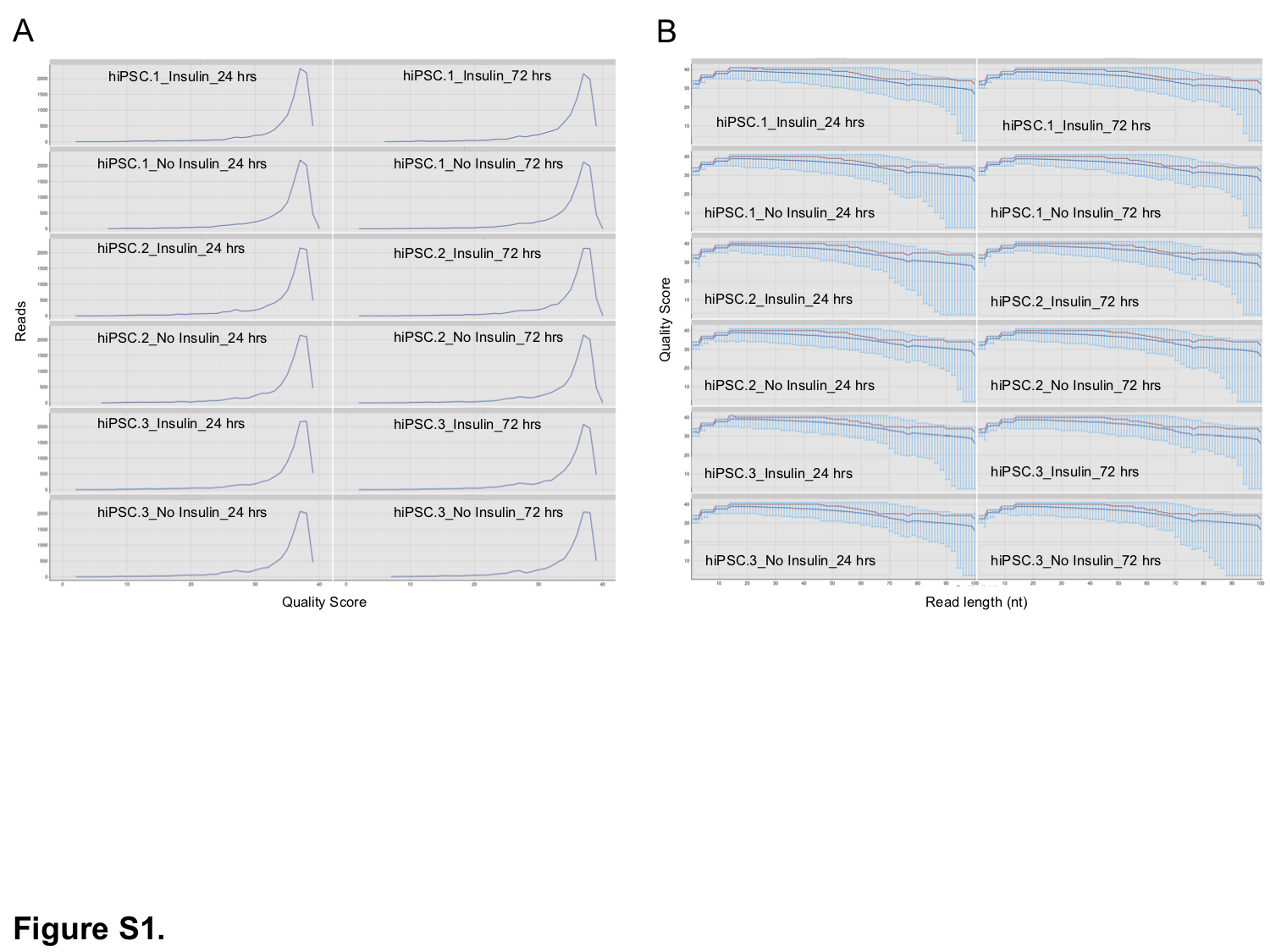

Supplement: Supplementary file 1 — Figure S1. RNA sequencing Data QC, related to results section regarding RNA sequencing. (A) Average read quality of each sequencing data. The x-axis shows the average read Q-score and the y-axis indicates the number of reads. (B) Base quality along the reads of the sequencing data. The x-axis shows the position in the read is plotted and the y-axis shows the Q-score is plotted. Median value Q-score are shown by the red lines. The mean value Q-score are represented by dark blue line. The inter-quartile range is shown by the boxplot, while the 10% and 90% points are represented by the whiskers. (TIFF 6593 kb) [file 13287_2019_1319_MOESM1_ESM.tiff]

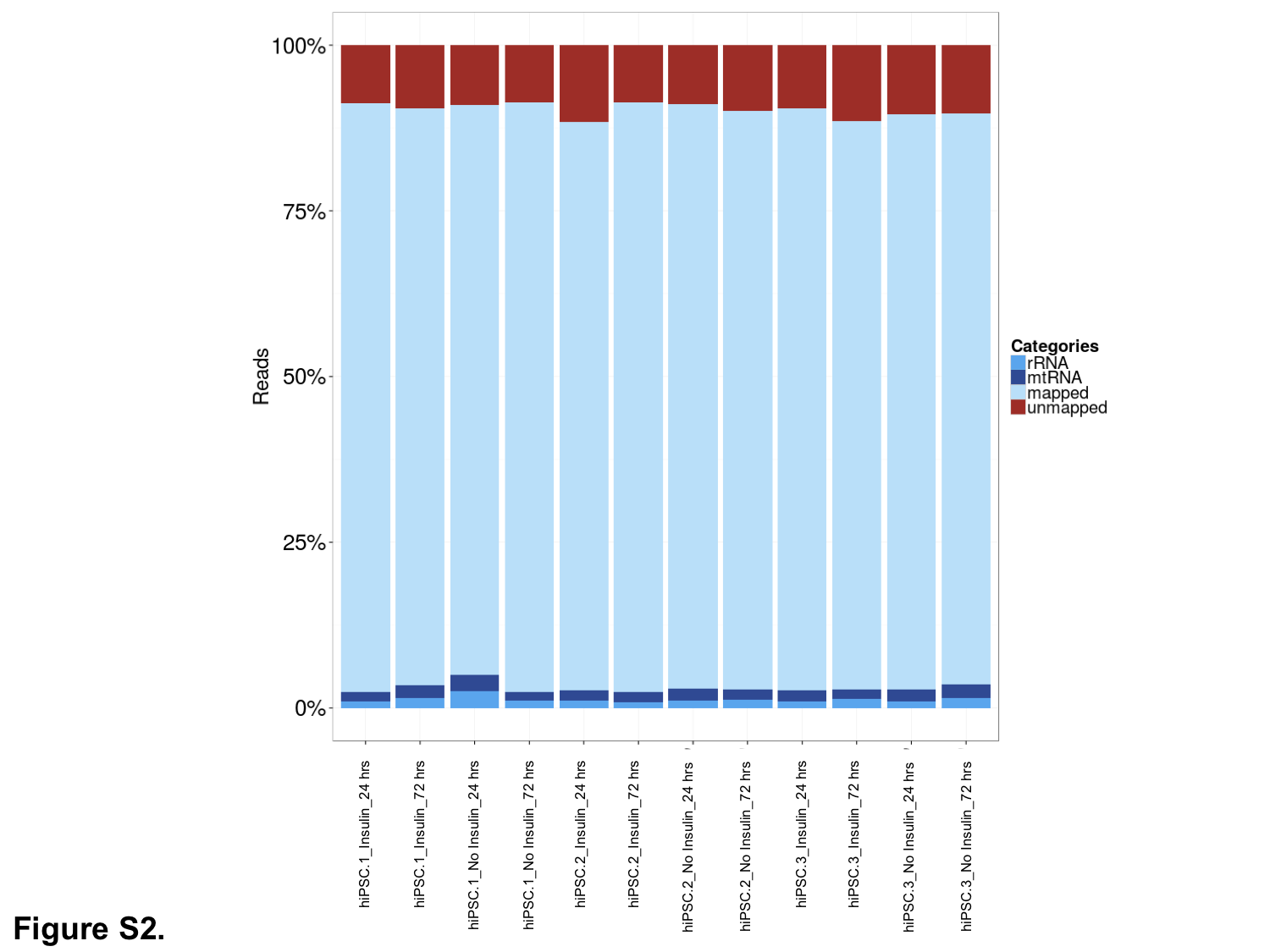

Supplement: Supplementary file 2 — Figure S2. Mapping results, related to the “Results” section regarding RNA sequencing. Samples’ summary of mapping results of the reads. In each sample reads could be classified into: mapped (aligned to reference genome), high abundance or out-mapped (e.g., rRNA, mtRNA, polyA, polyC) and unmapped (reads which did not align to anything). (TIFF 6593 kb) [file 13287_2019_1319_MOESM2_ESM.tiff]

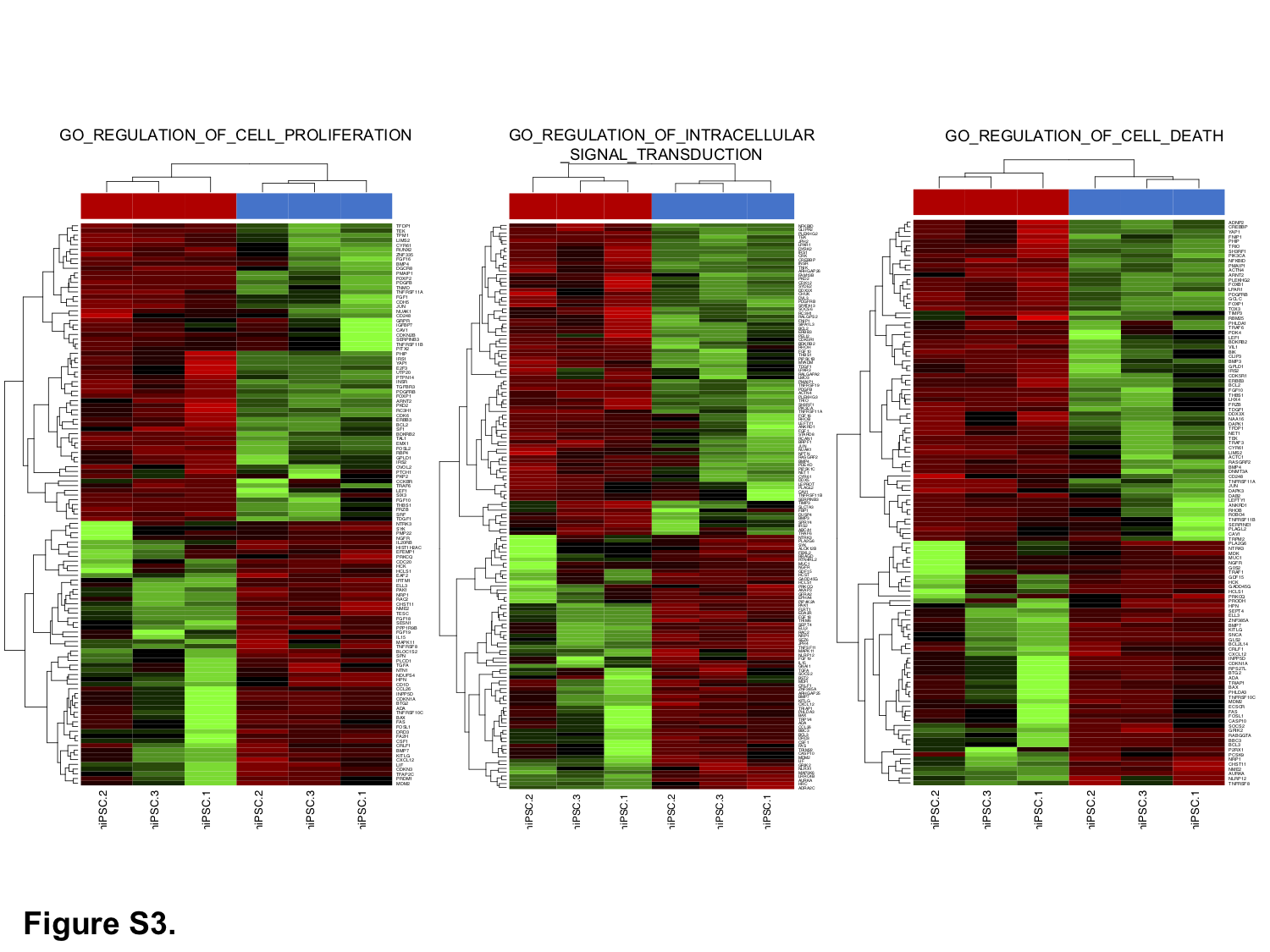

Supplement: Supplementary file 3 — Figure S3. Differentially expressed genes in selected GO pathways related to 24 h of hiPSC culture with or without insulin, related to Table 1 regarding the top 25 significant GO (biological process) terms. (TIFF 6593 kb) [file 13287_2019_1319_MOESM3_ESM.tiff]
