# Supplementary material for: The role of insulin as a key regulator of seeding, proliferation, and mRNA transcription of human pluripotent stem cells
Source: Stem Cell Res Ther. 2019 Jul 29;10:228. doi: 10.1186/s13287-019-1319-5 (PMC6664730; doi:10.1186/s13287-019-1319-5)
Supplement: Supplementary file 4 — Table S1. List of hiPSC lines used. Table S2. Number of identified genes in each sample, related to results section regarding RNA sequencing. Table S3. The 20 most significantly differentially expressed genes at 24 h of hiPSC culture with or without insulin, related to results section regarding RNA sequencing. The list is ordered by adjusted p values. Average read counts of samples with no insulin treatment are shown in the “baseMean” column. Fold change is the log2 fold change of normalized read count per gene between groups. Followed are the lfcSE (log fold change Standard Error) and stat (Wald statistics). Also listed are p values and adjusted p values corrected for multiple testing using the Benjamini-Hochberg False Discovery Rate (FDR) approach. Table S4. The 20 most significantly differentially expressed genes at 72 h of hiPSC culture with or without insulin, related to results section regarding RNA sequencing. The list is ordered by adjusted p values. Average read counts of samples with no insulin treatment are shown in the “baseMean” column. Fold change is the log2 fold change of normalized read count per gene between groups. Followed are the lfcSE (log fold change Standard Error) and stat (Wald statistics). Also listed are p values and adjusted p values corrected for multiple testing using the Benjamini-Hochberg False Discovery Rate (FDR) approach. Table S5. The top 100 significant GO (biological process) terms associated with differentially expressed transcripts after 72 h of hiPSC culture with or without insulin, related to results sections regarding RNA sequencing. Table S6. Differentially expressed genes from culture of hiPSCs with or without insulin that have been identified as a part of KEGG signaling pathways regulating pluripotency of stem cells (human). Table S7. Differentially expressed genes from culture of hiPSCs with or without insulin that have been identified as a part of KEGG insulin signaling pathway (human). (DOCX 74 kb) [file 13287_2019_1319_MOESM4_ESM.docx]

Table S1. List of hiPSC lines used.

| GENESIPS ID | Study ID | Age | Sex | Race |
| --- | --- | --- | --- | --- |
| 071-2 | hiPSC.1 | 55 | F | White |
| 334-1 | hiPSC.2 | 66 | F | White |
| 835-01 | hiPSC.3 | 64 | F | White |
| 756-03 | hiPSC.4 (Excluded) | 41 | F | White |

Table S2. Number of identified genes in each sample, related to results section regarding RNA sequencing.

| **Sample** | **Number of Genes identified** |
| --- | --- |
| hiPSC.1_No Insulin_24 hrs | 19,706 |
| hiPSC.2_No Insulin_24 hrs | 19,431 |
| hiPSC.3_No Insulin_24 hrs | 19,582 |
| hiPSC.1_Insulin_24 hrs | 19,252 |
| hiPSC.2_Insulin_24 hrs | 19,783 |
| hiPSC.3_Insulin_24 hrs | 19,659 |
| hiPSC.1_No Insulin_72 hrs | 19,569 |
| hiPSC.2_No Insulin_72 hrs | 19,604 |
| hiPSC.3_No Insulin_72 hrs | 19,221 |
| hiPSC.1_Insulin_72 hrs | 19,213 |
| hiPSC.2_Insulin_72 hrs | 19,348 |
| hiPSC.3_Insulin_72 hrs | 19,517 |

Table S3. The 20 most significantly differentially expressed genes at 24 hours of hiPSC culture with or without insulin.

| **GeneNames** | **baseMean** | **log2FoldChange** | **lfcSE** | **stat** | **p_value** | **p_adj** |
| --- | --- | --- | --- | --- | --- | --- |
| HPGD | 1027.98 | -5.17 | 0.32 | -16.19 | 5.51E-59 | 9.13E-55 |
| CDKN1A | 2806.23 | 3.90 | 0.28 | 13.71 | 9.32E-43 | 7.72E-39 |
| SEPP1 | 1037.21 | -3.14 | 0.26 | -12.02 | 2.93E-33 | 1.62E-29 |
| BMP7 | 1361.97 | 1.44 | 0.14 | 10.20 | 1.91E-24 | 6.75E-21 |
| PMAIP1 | 15060.09 | -1.78 | 0.17 | -10.20 | 2.04E-24 | 6.75E-21 |
| LYSMD4 | 1172.57 | -1.37 | 0.14 | -10.13 | 4.22E-24 | 1.16E-20 |
| EMX1 | 1038.46 | -1.93 | 0.20 | -9.53 | 1.55E-21 | 3.67E-18 |
| GPC4 | 36038.08 | -1.44 | 0.16 | -9.24 | 2.50E-20 | 5.18E-17 |
| ADAMTS17 | 328.46 | -2.53 | 0.28 | -9.20 | 3.42E-20 | 6.29E-17 |
| CCDC141 | 458.81 | -2.11 | 0.23 | -9.19 | 3.92E-20 | 6.50E-17 |
| MAP2 | 1774.66 | -2.56 | 0.28 | -9.12 | 7.62E-20 | 1.15E-16 |
| FGF1 | 215.25 | -3.37 | 0.37 | -9.09 | 9.65E-20 | 1.33E-16 |
| FOXP1 | 918.21 | -1.67 | 0.19 | -8.75 | 2.12E-18 | 2.71E-15 |
| TFAP2C | 466.46 | 1.69 | 0.19 | 8.74 | 2.36E-18 | 2.79E-15 |
| SHISA3 | 144.84 | 3.78 | 0.44 | 8.58 | 9.19E-18 | 1.01E-14 |
| PHLDA3 | 1482.73 | 1.80 | 0.21 | 8.38 | 5.48E-17 | 5.67E-14 |
| SESN1 | 1967.49 | 1.25 | 0.15 | 8.31 | 9.75E-17 | 9.49E-14 |
| PDGFB | 4113.70 | -2.79 | 0.34 | -8.17 | 2.96E-16 | 2.73E-13 |
| TNFRSF10C | 274.87 | 2.28 | 0.28 | 8.09 | 5.86E-16 | 4.96E-13 |
| LPAR1 | 228.21 | -1.86 | 0.23 | -8.09 | 5.99E-16 | 4.96E-13 |

Table S4. The 20 most significantly differentially expressed genes at 72 hours of hiPSC culture with or without insulin**.**

| **GeneNames** | **baseMean** | **log2FoldChange** | **lfcSE** | **stat** | **p_value** | **p_adj** |
| --- | --- | --- | --- | --- | --- | --- |
| MAP2 | 1774.66 | -5.54 | 0.29 | -19.25 | 1.48E-82 | 2.69E-78 |
| BMP7 | 1361.97 | 2.50 | 0.14 | 17.56 | 4.59E-69 | 4.18E-65 |
| VIL1 | 533.75 | -3.95 | 0.25 | -15.73 | 9.26E-56 | 5.62E-52 |
| LHFP | 401.98 | -3.36 | 0.22 | -15.61 | 6.38E-55 | 2.91E-51 |
| SERPINB4 | 266.11 | -5.30 | 0.36 | -14.56 | 4.69E-48 | 1.71E-44 |
| ISLR2 | 626.12 | 4.49 | 0.32 | 14.21 | 7.57E-46 | 2.30E-42 |
| TFAP2C | 466.46 | 2.63 | 0.19 | 13.86 | 1.05E-43 | 2.73E-40 |
| FGF1 | 215.25 | -5.61 | 0.41 | -13.58 | 4.93E-42 | 1.12E-38 |
| EML2 | 1690.78 | 2.45 | 0.18 | 13.57 | 6.30E-42 | 1.27E-38 |
| LYSMD4 | 1172.57 | -1.87 | 0.14 | -13.53 | 1.07E-41 | 1.95E-38 |
| CDKN1A | 2806.23 | 3.80 | 0.28 | 13.50 | 1.59E-41 | 2.63E-38 |
| TEK | 2531.86 | -4.43 | 0.33 | -13.47 | 2.39E-41 | 3.63E-38 |
| RGS5 | 2617.59 | -3.26 | 0.24 | -13.35 | 1.17E-40 | 1.64E-37 |
| PKI55 | 267.68 | -2.80 | 0.22 | -12.82 | 1.27E-37 | 1.65E-34 |
| CHST8 | 838.03 | 3.06 | 0.24 | 12.62 | 1.68E-36 | 2.05E-33 |
| PHYH | 2768.28 | 2.07 | 0.17 | 12.28 | 1.17E-34 | 1.34E-31 |
| TM4SF18 | 269.59 | -5.04 | 0.41 | -12.20 | 3.00E-34 | 3.22E-31 |
| GALNT11 | 2799.36 | 1.41 | 0.12 | 12.18 | 3.86E-34 | 3.90E-31 |
| AHCYL2 | 2152.25 | 1.91 | 0.16 | 12.12 | 8.78E-34 | 8.42E-31 |
| HCK | 246.13 | 4.78 | 0.40 | 11.88 | 1.44E-32 | 1.30E-29 |

Table S5. The top 100 significant GO (Biological Process) terms associated with differentially expressed transcripts after 72 hours of hiPSC culture with or without insulin.

| **-** | **GO Term** | **# Genes  in Gene Set (K)** | **# Genes  in Overlap (k)** | **k/K** | **p-value** | **FDR  q-value** |
| --- | --- | --- | --- | --- | --- | --- |
| 1 | GO_SMALL_MOLECULE_METABOLIC_PROCESS | 1767 | 304 | 0.172 | 1.37E-77 | 6.08E-74 |
| 2 | GO_PHOSPHATE_CONTAINING_COMPOUND_METABOLIC_PROCESS | 1977 | 315 | 0.1593 | 4.06E-72 | 9.00E-69 |
| 3 | GO_CELLULAR_RESPONSE_TO_ORGANIC_SUBSTANCE | 1848 | 297 | 0.1607 | 1.02E-68 | 1.50E-65 |
| 4 | GO_RESPONSE_TO_ENDOGENOUS_STIMULUS | 1450 | 248 | 0.171 | 2.00E-62 | 2.00E-59 |
| 5 | GO_ION_TRANSPORT | 1262 | 229 | 0.1815 | 2.26E-62 | 2.00E-59 |
| 6 | GO_ORGANONITROGEN_COMPOUND_METABOLIC_PROCESS | 1796 | 279 | 0.1553 | 3.13E-61 | 2.31E-58 |
| 7 | GO_REGULATION_OF_INTRACELLULAR_SIGNAL_TRANSDUCTION | 1656 | 260 | 0.157 | 6.97E-58 | 4.42E-55 |
| 8 | GO_CARBOHYDRATE_DERIVATIVE_METABOLIC_PROCESS | 1047 | 198 | 0.1891 | 7.95E-57 | 4.41E-54 |
| 9 | GO_TISSUE_DEVELOPMENT | 1518 | 242 | 0.1594 | 4.82E-55 | 2.38E-52 |
| 10 | GO_REGULATION_OF_TRANSPORT | 1804 | 268 | 0.1486 | 8.20E-55 | 3.64E-52 |
| 11 | GO_TRANSMEMBRANE_TRANSPORT | 1098 | 199 | 0.1812 | 4.74E-54 | 1.91E-51 |
| 12 | GO_REGULATION_OF_CELL_PROLIFERATION | 1496 | 231 | 0.1544 | 4.78E-50 | 1.77E-47 |
| 13 | GO_POSITIVE_REGULATION_OF_CELL_COMMUNICATION | 1532 | 232 | 0.1514 | 8.82E-49 | 3.01E-46 |
| 14 | GO_CELL_DEVELOPMENT | 1426 | 222 | 0.1557 | 1.11E-48 | 3.52E-46 |
| 15 | GO_REGULATION_OF_PHOSPHORUS_METABOLIC_PROCESS | 1618 | 239 | 0.1477 | 2.45E-48 | 7.24E-46 |
| 16 | GO_RESPONSE_TO_OXYGEN_CONTAINING_COMPOUND | 1381 | 215 | 0.1557 | 3.88E-47 | 1.08E-44 |
| 17 | GO_HOMEOSTATIC_PROCESS | 1337 | 210 | 0.1571 | 1.18E-46 | 3.08E-44 |
| 18 | GO_POSITIVE_REGULATION_OF_RESPONSE_TO_STIMULUS | 1929 | 263 | 0.1363 | 1.27E-46 | 3.13E-44 |
| 19 | GO_ORGANOPHOSPHATE_METABOLIC_PROCESS | 885 | 165 | 0.1864 | 1.59E-46 | 3.72E-44 |
| 20 | GO_PROTEIN_COMPLEX_SUBUNIT_ORGANIZATION | 1527 | 227 | 0.1487 | 2.36E-46 | 5.25E-44 |
| 21 | GO_PHOSPHORYLATION | 1228 | 198 | 0.1612 | 1.01E-45 | 2.13E-43 |
| 22 | GO_POSITIVE_REGULATION_OF_MULTICELLULAR_ORGANISMAL_PROCESS | 1395 | 213 | 0.1527 | 2.48E-45 | 4.99E-43 |
| 23 | GO_SINGLE_ORGANISM_BIOSYNTHETIC_PROCESS | 1340 | 206 | 0.1537 | 2.67E-44 | 5.15E-42 |
| 24 | GO_REGULATION_OF_PROTEIN_MODIFICATION_PROCESS | 1710 | 239 | 0.1398 | 3.83E-44 | 7.07E-42 |
| 25 | GO_REGULATION_OF_CELL_DIFFERENTIATION | 1492 | 219 | 0.1468 | 8.18E-44 | 1.41E-41 |
| 26 | GO_REGULATION_OF_MULTICELLULAR_ORGANISMAL_DEVELOPMENT | 1672 | 235 | 0.1406 | 8.29E-44 | 1.41E-41 |
| 27 | GO_LIPID_METABOLIC_PROCESS | 1158 | 187 | 0.1615 | 2.75E-43 | 4.52E-41 |
| 28 | GO_ION_TRANSMEMBRANE_TRANSPORT | 822 | 153 | 0.1861 | 4.36E-43 | 6.76E-41 |
| 29 | GO_CELLULAR_RESPONSE_TO_ENDOGENOUS_STIMULUS | 1008 | 172 | 0.1706 | 4.42E-43 | 6.76E-41 |
| 30 | GO_OXIDATION_REDUCTION_PROCESS | 898 | 160 | 0.1782 | 1.57E-42 | 2.31E-40 |
| 31 | GO_CATABOLIC_PROCESS | 1773 | 241 | 0.1359 | 1.92E-42 | 2.75E-40 |
| 32 | GO_CATION_TRANSPORT | 796 | 149 | 0.1872 | 2.78E-42 | 3.86E-40 |
| 33 | GO_REGULATION_OF_RESPONSE_TO_STRESS | 1468 | 213 | 0.1451 | 8.00E-42 | 1.08E-39 |
| 34 | GO_POSITIVE_REGULATION_OF_MOLECULAR_FUNCTION | 1791 | 241 | 0.1346 | 1.07E-41 | 1.39E-39 |
| 35 | GO_RESPONSE_TO_HORMONE | 893 | 158 | 0.1769 | 1.29E-41 | 1.63E-39 |
| 36 | GO_RESPONSE_TO_EXTERNAL_STIMULUS | 1821 | 242 | 0.1329 | 5.86E-41 | 7.22E-39 |
| 37 | GO_NEGATIVE_REGULATION_OF_RESPONSE_TO_STIMULUS | 1360 | 200 | 0.1471 | 3.94E-40 | 4.72E-38 |
| 38 | GO_CELLULAR_LIPID_METABOLIC_PROCESS | 913 | 157 | 0.172 | 8.62E-40 | 1.01E-37 |
| 39 | GO_NEUROGENESIS | 1402 | 203 | 0.1448 | 1.00E-39 | 1.14E-37 |
| 40 | GO_REGULATION_OF_HYDROLASE_ACTIVITY | 1327 | 196 | 0.1477 | 1.32E-39 | 1.47E-37 |
| 41 | GO_RESPONSE_TO_ORGANIC_CYCLIC_COMPOUND | 917 | 156 | 0.1701 | 5.84E-39 | 6.32E-37 |
| 42 | GO_CHEMICAL_HOMEOSTASIS | 874 | 150 | 0.1716 | 6.04E-38 | 6.38E-36 |
| 43 | GO_MOVEMENT_OF_CELL_OR_SUBCELLULAR_COMPONENT | 1275 | 188 | 0.1475 | 6.75E-38 | 6.96E-36 |
| 44 | GO_CIRCULATORY_SYSTEM_DEVELOPMENT | 788 | 141 | 0.1789 | 8.93E-38 | 9.00E-36 |
| 45 | GO_INTRACELLULAR_SIGNAL_TRANSDUCTION | 1572 | 214 | 0.1361 | 9.17E-38 | 9.04E-36 |
| 46 | GO_ORGANIC_ACID_METABOLIC_PROCESS | 953 | 157 | 0.1647 | 1.77E-37 | 1.71E-35 |
| 47 | GO_NEGATIVE_REGULATION_OF_CELL_COMMUNICATION | 1192 | 178 | 0.1493 | 1.26E-36 | 1.19E-34 |
| 48 | GO_SINGLE_ORGANISM_CATABOLIC_PROCESS | 957 | 155 | 0.162 | 4.06E-36 | 3.75E-34 |
| 49 | GO_POSITIVE_REGULATION_OF_BIOSYNTHETIC_PROCESS | 1805 | 230 | 0.1274 | 4.84E-36 | 4.38E-34 |
| 50 | GO_NUCLEOBASE_CONTAINING_SMALL_MOLECULE_METABOLIC_PROCESS | 535 | 111 | 0.2075 | 5.81E-36 | 5.15E-34 |
| 51 | GO_PROTEIN_COMPLEX_BIOGENESIS | 1132 | 171 | 0.1511 | 7.64E-36 | 6.65E-34 |
| 52 | GO_REGULATION_OF_CELL_DEATH | 1472 | 201 | 0.1365 | 1.15E-35 | 9.79E-34 |
| 53 | GO_POSITIVE_REGULATION_OF_GENE_EXPRESSION | 1733 | 223 | 0.1287 | 1.39E-35 | 1.16E-33 |
| 54 | GO_RESPONSE_TO_LIPID | 888 | 146 | 0.1644 | 8.34E-35 | 6.85E-33 |
| 55 | GO_POSITIVE_REGULATION_OF_CATALYTIC_ACTIVITY | 1518 | 203 | 0.1337 | 9.85E-35 | 7.95E-33 |
| 56 | GO_RESPONSE_TO_NITROGEN_COMPOUND | 859 | 143 | 0.1665 | 1.03E-34 | 8.19E-33 |
| 57 | GO_PROTEIN_LOCALIZATION | 1805 | 227 | 0.1258 | 1.05E-34 | 8.21E-33 |
| 58 | GO_MACROMOLECULAR_COMPLEX_ASSEMBLY | 1398 | 191 | 0.1366 | 6.12E-34 | 4.68E-32 |
| 59 | GO_REGULATION_OF_ANATOMICAL_STRUCTURE_MORPHOGENESIS | 1021 | 157 | 0.1538 | 7.18E-34 | 5.40E-32 |
| 60 | GO_REGULATION_OF_CELLULAR_COMPONENT_MOVEMENT | 771 | 132 | 0.1712 | 2.21E-33 | 1.63E-31 |
| 61 | GO_POSITIVE_REGULATION_OF_DEVELOPMENTAL_PROCESS | 1142 | 167 | 0.1462 | 2.80E-33 | 2.04E-31 |
| 62 | GO_ORGAN_MORPHOGENESIS | 841 | 137 | 0.1629 | 2.76E-32 | 1.98E-30 |
| 63 | GO_REGULATION_OF_CELLULAR_LOCALIZATION | 1277 | 177 | 0.1386 | 2.86E-32 | 2.02E-30 |
| 64 | GO_POSITIVE_REGULATION_OF_PROTEIN_METABOLIC_PROCESS | 1492 | 195 | 0.1307 | 4.85E-32 | 3.36E-30 |
| 65 | GO_ION_HOMEOSTASIS | 576 | 109 | 0.1892 | 1.29E-31 | 8.78E-30 |
| 66 | GO_POSITIVE_REGULATION_OF_PHOSPHORUS_METABOLIC_PROCESS | 1036 | 154 | 0.1486 | 1.51E-31 | 1.01E-29 |
| 67 | GO_CELLULAR_RESPONSE_TO_STRESS | 1565 | 200 | 0.1278 | 1.57E-31 | 1.04E-29 |
| 68 | GO_IMMUNE_SYSTEM_PROCESS | 1984 | 234 | 0.1179 | 1.59E-31 | 1.04E-29 |
| 69 | GO_GLYCOSYL_COMPOUND_METABOLIC_PROCESS | 368 | 85 | 0.231 | 2.35E-31 | 1.51E-29 |
| 70 | GO_REGULATION_OF_TRANSCRIPTION_FROM_RNA_POLYMERASE_II_PROMOTER | 1784 | 217 | 0.1216 | 4.13E-31 | 2.62E-29 |
| 71 | GO_NEGATIVE_REGULATION_OF_MULTICELLULAR_ORGANISMAL_PROCESS | 983 | 148 | 0.1506 | 5.84E-31 | 3.65E-29 |
| 72 | GO_EPITHELIUM_DEVELOPMENT | 945 | 144 | 0.1524 | 1.05E-30 | 6.44E-29 |
| 73 | GO_POSITIVE_REGULATION_OF_TRANSPORT | 936 | 143 | 0.1528 | 1.27E-30 | 7.75E-29 |
| 74 | GO_RESPONSE_TO_ABIOTIC_STIMULUS | 1024 | 151 | 0.1475 | 1.46E-30 | 8.73E-29 |
| 75 | GO_INORGANIC_ION_TRANSMEMBRANE_TRANSPORT | 583 | 108 | 0.1852 | 1.65E-30 | 9.78E-29 |
| 76 | GO_POSITIVE_REGULATION_OF_INTRACELLULAR_SIGNAL_TRANSDUCTION | 876 | 137 | 0.1564 | 2.01E-30 | 1.17E-28 |
| 77 | GO_CELLULAR_CATABOLIC_PROCESS | 1322 | 177 | 0.1339 | 2.10E-30 | 1.21E-28 |
| 78 | GO_CYTOSKELETON_ORGANIZATION | 838 | 133 | 0.1587 | 3.27E-30 | 1.86E-28 |
| 79 | GO_NEGATIVE_REGULATION_OF_GENE_EXPRESSION | 1493 | 191 | 0.1279 | 3.43E-30 | 1.93E-28 |
| 80 | GO_ESTABLISHMENT_OF_LOCALIZATION_IN_CELL | 1676 | 206 | 0.1229 | 3.72E-30 | 2.06E-28 |
| 81 | GO_CELLULAR_HOMEOSTASIS | 676 | 117 | 0.1731 | 3.80E-30 | 2.08E-28 |
| 82 | GO_POSITIVE_REGULATION_OF_HYDROLASE_ACTIVITY | 905 | 139 | 0.1536 | 4.96E-30 | 2.68E-28 |
| 83 | GO_PURINE_CONTAINING_COMPOUND_METABOLIC_PROCESS | 394 | 86 | 0.2183 | 8.33E-30 | 4.45E-28 |
| 84 | GO_CELLULAR_COMPONENT_MORPHOGENESIS | 900 | 138 | 0.1533 | 9.56E-30 | 5.05E-28 |
| 85 | GO_REGULATION_OF_ION_TRANSPORT | 592 | 107 | 0.1807 | 2.69E-29 | 1.41E-27 |
| 86 | GO_NEGATIVE_REGULATION_OF_PROTEIN_METABOLIC_PROCESS | 1087 | 154 | 0.1417 | 3.27E-29 | 1.69E-27 |
| 87 | GO_LOCOMOTION | 1114 | 156 | 0.14 | 5.11E-29 | 2.60E-27 |
| 88 | GO_CELLULAR_CHEMICAL_HOMEOSTASIS | 570 | 104 | 0.1825 | 7.40E-29 | 3.73E-27 |
| 89 | GO_NEGATIVE_REGULATION_OF_DEVELOPMENTAL_PROCESS | 801 | 127 | 0.1586 | 7.49E-29 | 3.73E-27 |
| 90 | GO_REGULATION_OF_MAPK_CASCADE | 660 | 113 | 0.1712 | 9.89E-29 | 4.87E-27 |
| 91 | GO_ANATOMICAL_STRUCTURE_FORMATION_INVOLVED_IN_MORPHOGENESIS | 957 | 141 | 0.1473 | 1.47E-28 | 7.15E-27 |
| 92 | GO_CELLULAR_RESPONSE_TO_OXYGEN_CONTAINING_COMPOUND | 799 | 126 | 0.1577 | 2.08E-28 | 1.00E-26 |
| 93 | GO_ENZYME_LINKED_RECEPTOR_PROTEIN_SIGNALING_PATHWAY | 689 | 115 | 0.1669 | 3.24E-28 | 1.55E-26 |
| 94 | GO_CENTRAL_NERVOUS_SYSTEM_DEVELOPMENT | 872 | 132 | 0.1514 | 6.04E-28 | 2.85E-26 |
| 95 | GO_CARBOHYDRATE_DERIVATIVE_BIOSYNTHETIC_PROCESS | 595 | 105 | 0.1765 | 6.93E-28 | 3.24E-26 |
| 96 | GO_CELL_CYCLE | 1316 | 171 | 0.1299 | 7.11E-28 | 3.28E-26 |
| 97 | GO_REGULATION_OF_ORGANELLE_ORGANIZATION | 1178 | 159 | 0.135 | 8.97E-28 | 4.10E-26 |
| 98 | GO_NEGATIVE_REGULATION_OF_NITROGEN_COMPOUND_METABOLIC_PROCESS | 1517 | 187 | 0.1233 | 1.42E-27 | 6.44E-26 |
| 99 | GO_METAL_ION_TRANSPORT | 582 | 103 | 0.177 | 1.76E-27 | 7.88E-26 |
| 100 | GO_REGULATION_OF_CELL_DEVELOPMENT | 836 | 127 | 0.1519 | 4.51E-27 | 2.00E-25 |

Table S6. Differentially expressed genes from culture of hiPSCs with or without insulin that have been identified as a part of KEGG signaling pathways regulating pluripotency of stem cells (human).

| **Time** | **#** | **Gene_Symbol** | **baseMean** | **log2FoldChange** | **lfcSE** | **stat** | **p_value** | **p_adj** |
| --- | --- | --- | --- | --- | --- | --- | --- | --- |
| 24hrs | 1 | LIF | 431.90 | 1.01 | 0.26 | 3.86 | 1.15E-04 | 4.34E-03 |
|  | 2 | PIK3CA.1 | 725.49 | -0.75 | 0.18 | -4.16 | 3.21E-05 | 1.60E-03 |
|  | 3 | BMP4 | 488.36 | -2.39 | 0.67 | -3.55 | 3.87E-04 | 1.11E-02 |
|  | 4 | MAPK11 | 148.75 | 0.88 | 0.29 | 3.07 | 2.13E-03 | 3.59E-02 |
|  | 5 | FZD2 | 3190.69 | -0.65 | 0.22 | -2.96 | 3.03E-03 | 4.52E-02 |
|  | 6 | DVL3 | 5369.13 | -0.54 | 0.13 | -4.12 | 3.78E-05 | 1.80E-03 |
|  | 7 | APC2 | 209.57 | 1.10 | 0.33 | 3.33 | 8.79E-04 | 1.98E-02 |
| 72hrs | 1 | LIF | 431.90 | 1.23 | 0.26 | 4.72 | 2.36E-06 | 2.56E-05 |
|  | 2 | JAK3 | 573.38 | 0.71 | 0.20 | 3.49 | 4.75E-04 | 2.37E-03 |
|  | 3 | STAT3 | 6512.86 | 0.51 | 0.14 | 3.68 | 2.35E-04 | 1.32E-03 |
|  | 4 | KLF4 | 136.36 | 0.96 | 0.27 | 3.56 | 3.73E-04 | 1.93E-03 |
|  | 5 | MYC | 5078.23 | -1.44 | 0.30 | -4.73 | 2.22E-06 | 2.43E-05 |
|  | 6 | PIK3CA.1 | 725.49 | -0.63 | 0.18 | -3.53 | 4.16E-04 | 2.12E-03 |
|  | 7 | PIK3CD.1 | 2228.94 | -0.99 | 0.24 | -4.17 | 3.04E-05 | 2.34E-04 |
|  | 8 | PIK3R2.1 | 4542.85 | -0.73 | 0.18 | -4.17 | 3.08E-05 | 2.37E-04 |
|  | 9 | AKT1.1 | 5547.49 | -0.50 | 0.16 | -3.07 | 2.14E-03 | 8.42E-03 |
|  | 10 | AKT2.1 | 6796.97 | -0.37 | 0.13 | -2.72 | 6.52E-03 | 2.12E-02 |
|  | 11 | INHBA | 25.68 | -4.59 | 0.93 | -4.96 | 6.95E-07 | 8.72E-06 |
|  | 12 | INHBC | 44.11 | 1.79 | 0.70 | 2.56 | 1.06E-02 | 3.16E-02 |
|  | 13 | ACVR1B | 4477.23 | 1.04 | 0.28 | 3.69 | 2.28E-04 | 1.29E-03 |
|  | 14 | ACVR1C | 107.66 | 1.49 | 0.32 | 4.58 | 4.56E-06 | 4.56E-05 |
|  | 15 | BMP4 | 488.36 | -2.34 | 0.67 | -3.49 | 4.92E-04 | 2.44E-03 |
|  | 16 | ACVR1 | 1329.75 | 1.69 | 0.28 | 5.97 | 2.36E-09 | 5.38E-08 |
|  | 17 | SMAD9 | 103.47 | -1.28 | 0.38 | -3.33 | 8.68E-04 | 3.96E-03 |
|  | 18 | ID3 | 4887.32 | -1.02 | 0.28 | -3.66 | 2.53E-04 | 1.40E-03 |
|  | 19 | MAPK11 | 148.75 | 1.96 | 0.28 | 6.92 | 4.47E-12 | 1.85E-10 |
|  | 20 | MAPK13 | 1712.11 | 0.66 | 0.26 | 2.52 | 1.17E-02 | 3.45E-02 |
|  | 21 | WNT2B | 27.06 | -2.07 | 0.57 | -3.62 | 2.94E-04 | 1.59E-03 |
|  | 22 | WNT16 | 8.22 | -3.12 | 1.22 | -2.56 | 1.05E-02 | 3.15E-02 |
|  | 23 | FZD7 | 11336.08 | -1.18 | 0.25 | -4.82 | 1.44E-06 | 1.65E-05 |
|  | 24 | FZD2 | 3190.69 | -0.91 | 0.22 | -4.13 | 3.56E-05 | 2.67E-04 |
|  | 25 | DVL3 | 5369.13 | -0.71 | 0.13 | -5.41 | 6.36E-08 | 1.04E-06 |
|  | 26 | AXIN1 | 4608.11 | -0.44 | 0.12 | -3.73 | 1.91E-04 | 1.11E-03 |
|  | 27 | APC | 1540.13 | -0.67 | 0.28 | -2.43 | 1.53E-02 | 4.28E-02 |
|  | 28 | APC2 | 209.57 | 1.29 | 0.33 | 3.90 | 9.51E-05 | 6.11E-04 |
|  | 29 | TCF3 | 18038.84 | -0.49 | 0.16 | -2.95 | 3.13E-03 | 1.16E-02 |
|  | 30 | ESRRB | 101.35 | 1.73 | 0.46 | 3.75 | 1.80E-04 | 1.05E-03 |
|  | 31 | HNF1A | 46.57 | -1.99 | 0.43 | -4.62 | 3.84E-06 | 3.92E-05 |
|  | 32 | HESX1 | 134.81 | 0.98 | 0.40 | 2.46 | 1.38E-02 | 3.94E-02 |
|  | 33 | SMARCAD1 | 4212.83 | -0.98 | 0.21 | -4.62 | 3.90E-06 | 3.97E-05 |
|  | 34 | RIF1 | 5456.64 | -0.60 | 0.23 | -2.61 | 9.17E-03 | 2.81E-02 |
|  | 35 | PCGF1 | 378.01 | 0.63 | 0.21 | 2.95 | 3.14E-03 | 1.17E-02 |
|  | 36 | PCGF3 | 3161.25 | -0.66 | 0.15 | -4.27 | 1.98E-05 | 1.63E-04 |
|  | 37 | LHX5 | 77.28 | -3.50 | 1.30 | -2.70 | 6.90E-03 | 2.23E-02 |
|  | 38 | ONECUT1 | 86.97 | 1.75 | 0.64 | 2.75 | 5.92E-03 | 1.96E-02 |
|  | 39 | LEFTY2 | 1722.43 | -2.95 | 0.71 | -4.14 | 3.48E-05 | 2.62E-04 |

Table S7. Differentially expressed genes from culture of hiPSCs with or without insulin that have been identified as a part of KEGG insulin signaling pathway (human).

| **Time** | **#** | **Gene_Symbol** | **baseMean** | **log2FoldChange** | **lfcSE** | **stat** | **p_value** | **p_adj** |
| --- | --- | --- | --- | --- | --- | --- | --- | --- |
| 24hr | 1 | IRS4 | 557.11 | -1.25 | 0.21 | -5.99 | 2.04E-09 | 4.23E-07 |
|  | 2 | INSR | 2832.53 | -0.80 | 0.17 | -4.85 | 1.23E-06 | 1.04E-04 |
|  | 3 | PIK3CA | 725.49 | -0.75 | 0.18 | -4.16 | 3.21E-05 | 1.60E-03 |
|  | 4 | LIPE | 787.18 | 0.76 | 0.18 | 4.15 | 3.38E-05 | 1.66E-03 |
|  | 5 | IRS2 | 2499.16 | -0.97 | 0.25 | -3.84 | 1.24E-04 | 4.55E-03 |
|  | 6 | FBP1 | 244.77 | -1.20 | 0.31 | -3.84 | 1.24E-04 | 4.55E-03 |
|  | 7 | SOCS2 | 1172.46 | 1.16 | 0.32 | 3.67 | 2.39E-04 | 7.70E-03 |
|  | 8 | IRS1 | 3539.76 | -0.78 | 0.23 | -3.33 | 8.54E-04 | 1.94E-02 |
|  | 9 | CRK | 329.30 | -0.64 | 0.21 | -3.13 | 1.72E-03 | 3.15E-02 |
| 72hr | 1 | PRKACB | 1805.12 | -1.39 | 0.21 | -6.66 | 2.78E-11 | 9.92E-10 |
|  | 2 | IRS4 | 557.11 | -1.39 | 0.21 | -6.63 | 3.29E-11 | 1.16E-09 |
|  | 3 | PRKAB2 | 3105.28 | 1.00 | 0.16 | 6.34 | 2.32E-10 | 6.82E-09 |
|  | 4 | PRKAB1 | 2323.36 | 0.95 | 0.17 | 5.64 | 1.72E-08 | 3.26E-07 |
|  | 5 | FASN | 52376.95 | -1.17 | 0.22 | -5.28 | 1.26E-07 | 1.92E-06 |
|  | 6 | PRKAG2 | 741.05 | -1.21 | 0.23 | -5.19 | 2.10E-07 | 3.03E-06 |
|  | 7 | GCK | 269.45 | -1.63 | 0.32 | -5.02 | 5.21E-07 | 6.84E-06 |
|  | 8 | SHC1 | 23122.40 | 0.84 | 0.17 | 5.00 | 5.75E-07 | 7.46E-06 |
|  | 9 | RAPGEF1 | 7845.64 | -0.69 | 0.14 | -4.83 | 1.38E-06 | 1.60E-05 |
|  | 10 | IRS1 | 3539.76 | -1.09 | 0.23 | -4.67 | 2.97E-06 | 3.14E-05 |
|  | 11 | HKDC1 | 13.73 | -5.24 | 1.17 | -4.50 | 6.78E-06 | 6.50E-05 |
|  | 12 | TRIP10 | 5247.86 | 0.83 | 0.19 | 4.43 | 9.51E-06 | 8.71E-05 |
|  | 13 | FLOT2 | 12408.07 | 0.58 | 0.13 | 4.41 | 1.02E-05 | 9.20E-05 |
|  | 14 | SOCS1 | 965.39 | 0.97 | 0.22 | 4.39 | 1.12E-05 | 1.00E-04 |
|  | 15 | PPP1R3E | 273.94 | 1.48 | 0.35 | 4.18 | 2.86E-05 | 2.23E-04 |
|  | 16 | PIK3CD | 2228.94 | -0.99 | 0.24 | -4.17 | 3.04E-05 | 2.34E-04 |
|  | 17 | PIK3R2 | 4542.85 | -0.73 | 0.18 | -4.17 | 3.08E-05 | 2.37E-04 |
|  | 18 | RHEB | 5428.55 | 0.67 | 0.17 | 4.07 | 4.70E-05 | 3.38E-04 |
|  | 19 | INPPL1 | 5756.72 | -0.72 | 0.18 | -4.03 | 5.51E-05 | 3.88E-04 |
|  | 20 | INSR | 2832.53 | -0.66 | 0.17 | -3.98 | 6.77E-05 | 4.61E-04 |
|  | 21 | LIPE | 787.18 | 0.69 | 0.18 | 3.76 | 1.68E-04 | 9.91E-04 |
|  | 22 | PHKA1 | 844.14 | 0.64 | 0.17 | 3.72 | 1.99E-04 | 1.15E-03 |
|  | 23 | MAPK8 | 2966.31 | -0.54 | 0.15 | -3.57 | 3.60E-04 | 1.88E-03 |
|  | 24 | PIK3CA | 725.49 | -0.63 | 0.18 | -3.53 | 4.16E-04 | 2.12E-03 |
|  | 25 | PRKAG1 | 2072.35 | 0.65 | 0.19 | 3.45 | 5.66E-04 | 2.75E-03 |
|  | 26 | CALM1 | 15065.99 | -0.42 | 0.12 | -3.45 | 5.67E-04 | 2.76E-03 |
|  | 27 | FBP1 | 244.77 | -1.04 | 0.31 | -3.38 | 7.16E-04 | 3.37E-03 |
|  | 28 | PYGB | 5945.72 | -0.48 | 0.14 | -3.37 | 7.49E-04 | 3.50E-03 |
|  | 29 | PPP1CC | 17593.65 | -0.41 | 0.12 | -3.34 | 8.45E-04 | 3.87E-03 |
|  | 30 | MKNK1 | 517.52 | -0.59 | 0.18 | -3.34 | 8.52E-04 | 3.90E-03 |
|  | 31 | PRKAR1A | 10213.97 | 0.43 | 0.13 | 3.25 | 1.17E-03 | 5.09E-03 |
|  | 32 | PPP1R3B | 2658.67 | -0.93 | 0.29 | -3.22 | 1.27E-03 | 5.49E-03 |
|  | 33 | CRK | 329.30 | -0.65 | 0.20 | -3.16 | 1.57E-03 | 6.52E-03 |
|  | 34 | PPP1CB | 7968.36 | -0.41 | 0.13 | -3.13 | 1.77E-03 | 7.18E-03 |
|  | 35 | SOCS3 | 1593.28 | -1.07 | 0.34 | -3.11 | 1.86E-03 | 7.48E-03 |
|  | 36 | AKT1 | 5547.49 | -0.50 | 0.16 | -3.07 | 2.14E-03 | 8.42E-03 |
|  | 37 | SHC3 | 352.29 | -1.02 | 0.34 | -3.01 | 2.64E-03 | 1.00E-02 |
|  | 38 | SOCS2 | 1172.46 | 0.91 | 0.32 | 2.87 | 4.07E-03 | 1.44E-02 |
|  | 39 | EXOC7 | 5328.99 | -0.39 | 0.14 | -2.81 | 4.96E-03 | 1.69E-02 |
|  | 40 | AKT2 | 6796.97 | -0.37 | 0.13 | -2.72 | 6.52E-03 | 2.12E-02 |
|  | 41 | EIF4E | 3622.24 | 0.35 | 0.13 | 2.65 | 8.09E-03 | 2.54E-02 |
|  | 42 | SORBS1 | 2161.11 | -0.41 | 0.15 | -2.63 | 8.58E-03 | 2.67E-02 |
|  | 43 | MAPK10 | 110.76 | -0.92 | 0.36 | -2.58 | 9.89E-03 | 3.00E-02 |
|  | 44 | ELK1 | 2714.94 | -0.36 | 0.15 | -2.39 | 1.70E-02 | 4.67E-02 |
